# Supplementary material for: Chemoenzymatic Syntheses of Fluorine-18-Labeled Disaccharides from [18F] FDG Yield Potent Sensors of Living Bacteria In Vivo
Source: J Am Chem Soc. 2023 Aug 3;145(32):17632–42. doi: 10.1021/jacs.3c03338 (PMC10436271; doi:10.1021/jacs.3c03338)
Supplement: Supplementary file 1 — ja3c03338_si_001.pdf [file ja3c03338_si_001.pdf]

Supporting Information for:

<u>Chemoenzymatic syntheses of fluorine-18-labeled disaccharides from [^{18}F]FDG yield potent sensors of living bacteria *in vivo*</u>

Alexandre M. Sorlin[1], Marina López-Álvarez[1], Sarah J. Rabbitt[1], Aryn A. Alanizi[1], Rebecca Shuere[1], Kondapa Naidu Bobba[1], Joseph Blecha[1], Sasank Sakhamuri[1], Michael J. Evans[1], Kenneth W. Bayles[2], Robert R. Flavell[1], Oren S. Rosenberg[3], Renuka Sriram[4], Tom Desmet[4], Bernd Nidetzky[5], Joanne Engel[4], Michael A. Ohliger[1,6], James S. Fraser[7], David M. Wilson[1*]

[1]Department of Radiology and Biomedical Imaging
University of California, San Francisco
San Francisco, CA 94158, USA

[2]Department of Pathology and Microbiology
University of Nebraska Medical Center
Omaha, NE 68198, USA

[3]Department of Medicine
University of California, San Francisco
San Francisco, CA 94158, USA

[4]Department of Biotechnology
Ghent University
Gent, Belgium

[5]Institute of Biotechnology and Biochemical Engineering
Graz University of Technology
Graz, Austria

[6]Department of Radiology
Zuckerberg San Francisco General Hospital
San Francisco, CA 94110, USA

[7]Department of Bioengineering and Therapeutic Sciences
University of California, San Francisco
San Francisco, CA 94158, USA

Table of Contents

A. **Supplemental Figures……………………………………………………………………2**
B. **Synthetic Procedures…………………………………………………………………20**
C. **Radiochemistry………………………………………………………………………..43**
D. **References……………………………………………………………………………….56**

A. Supplemental Figures

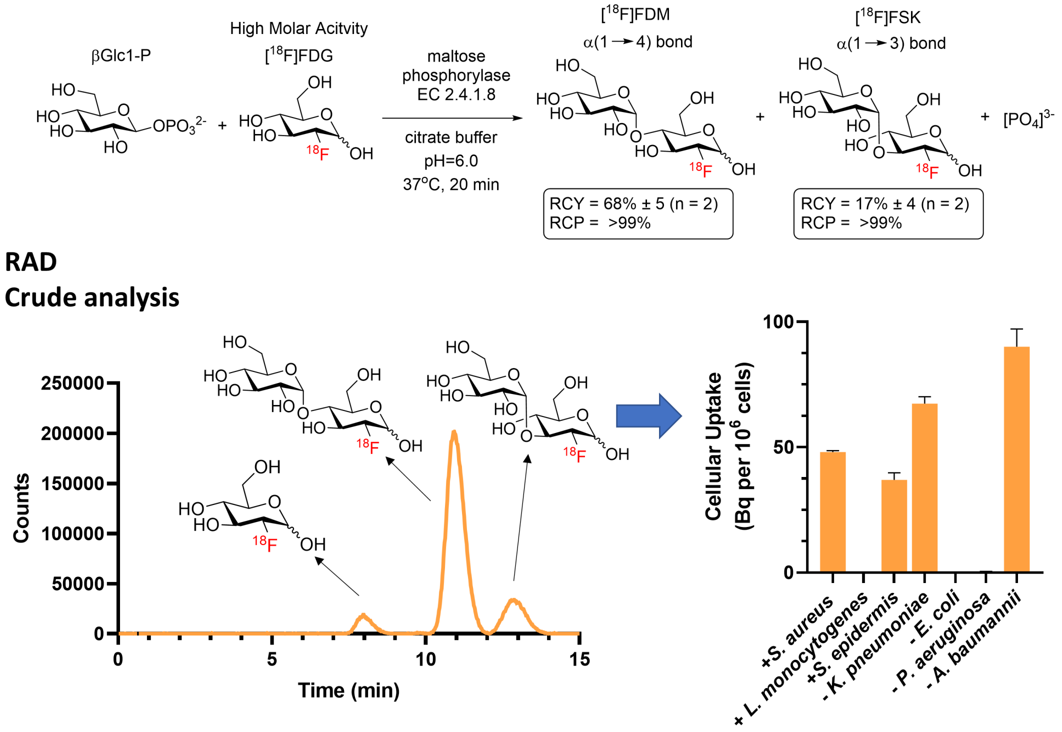

Figure S1. Radiosynthesis of [^{18}F]FSK using "high molar activity" [^{18}F]FDG and *in vitro* uptake.

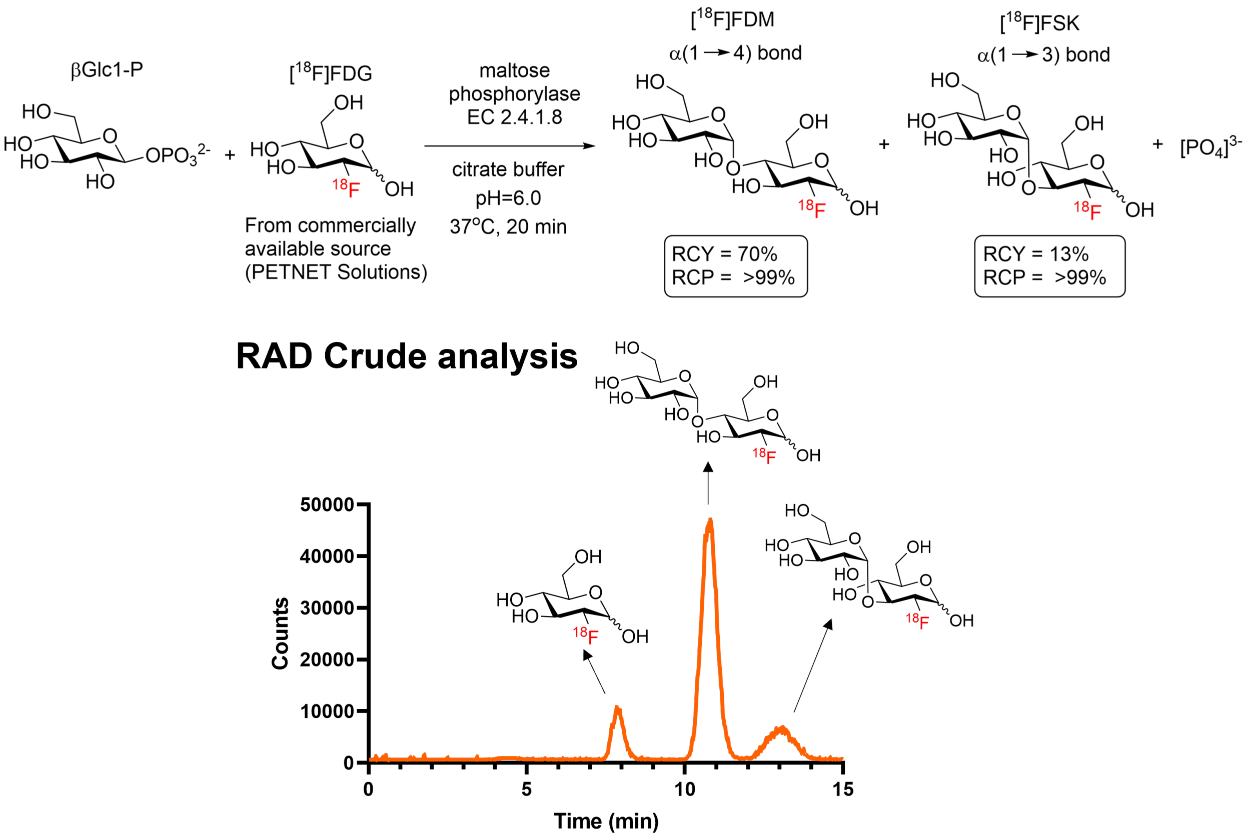

Figure S2. Radiosynthesis of [^{18}F]FDM and [^{18}F]FSK using commercially available [^{18}F]FDG (PETNET solutions).

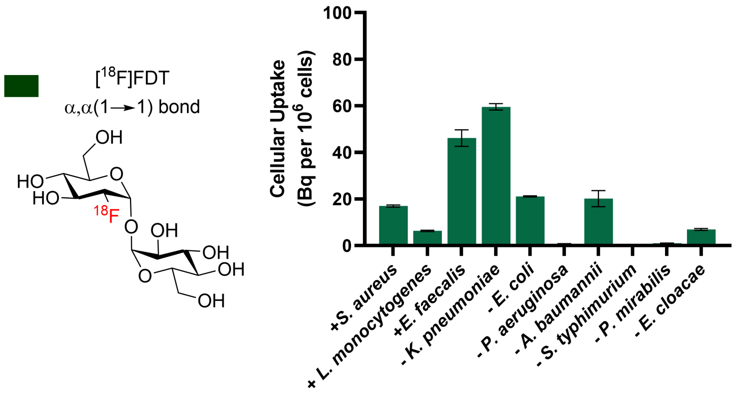

Figure S3. *In vitro* uptake of [^{18}F]FDT in *S. aureus, L. monocytogenes, E. faecalis, K. pneumoniae, E. coli, P. aeruginosa, A. baumannii, S. typhimurium, P. mirabilis, E. cloacae*.

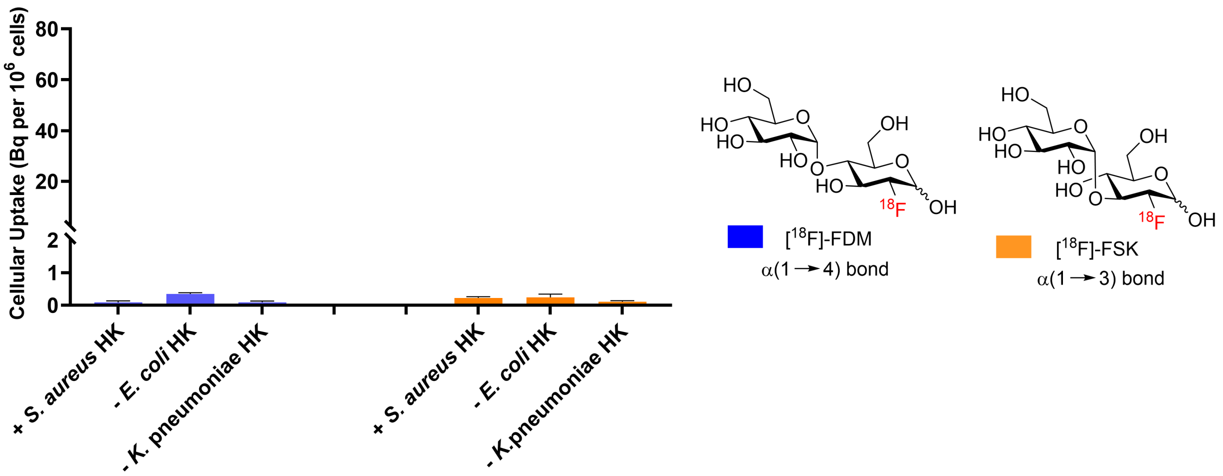

Figure S4. *In vitro* uptake of [^{18}F]FDM and [^{18}F]FSK in heat-killed bacteria.

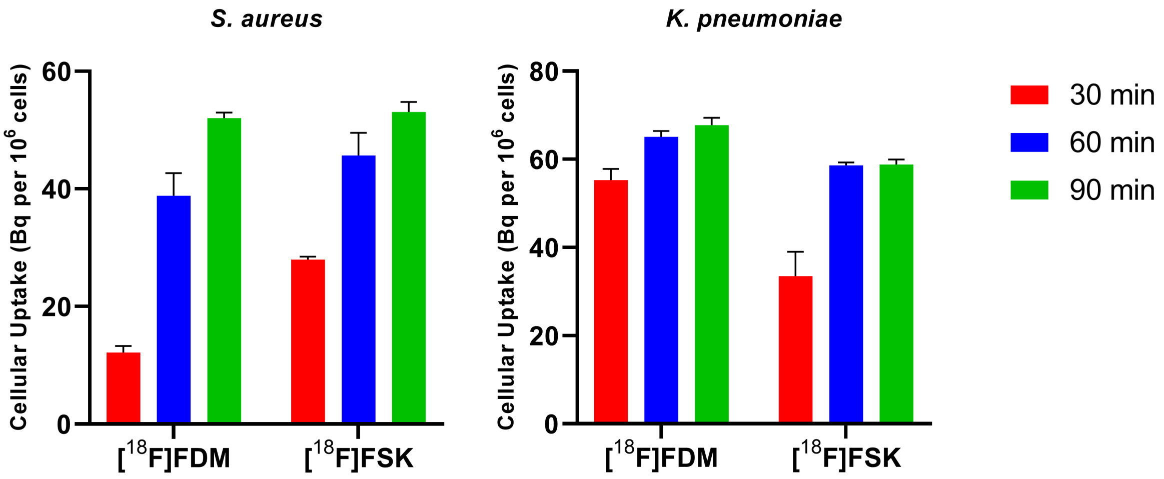

Figure S5. *In vitro* uptake of [^{18}F]FDM and [^{18}F]FSK in *S. aureus* and *K. pneumoniae* at 30 min, 60 min and 90 min.

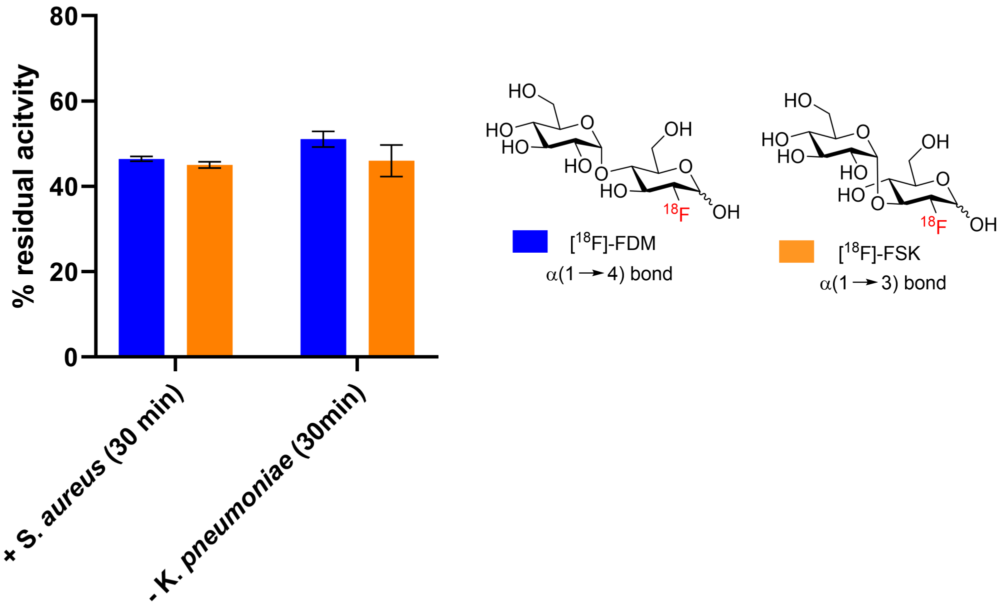

Figure S6. Efflux experiment: plot showing the residual activity observed at 30 min post efflux of [^{18}F]FDM and [^{18}F]FSK in *S. aureus* and *K. pneumoniae* following an initial 30 min incubation.

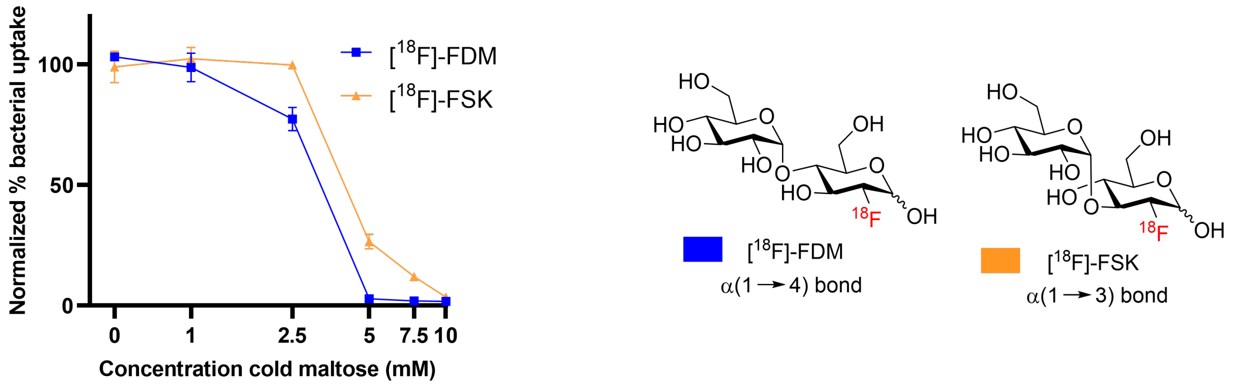

Figure S7. Cross blocking experiment: competition of [^{18}F]FDM and [^{18}F]FSK uptake with increasing concentrations of unlabeled (cold) maltose in *S. aureus*.

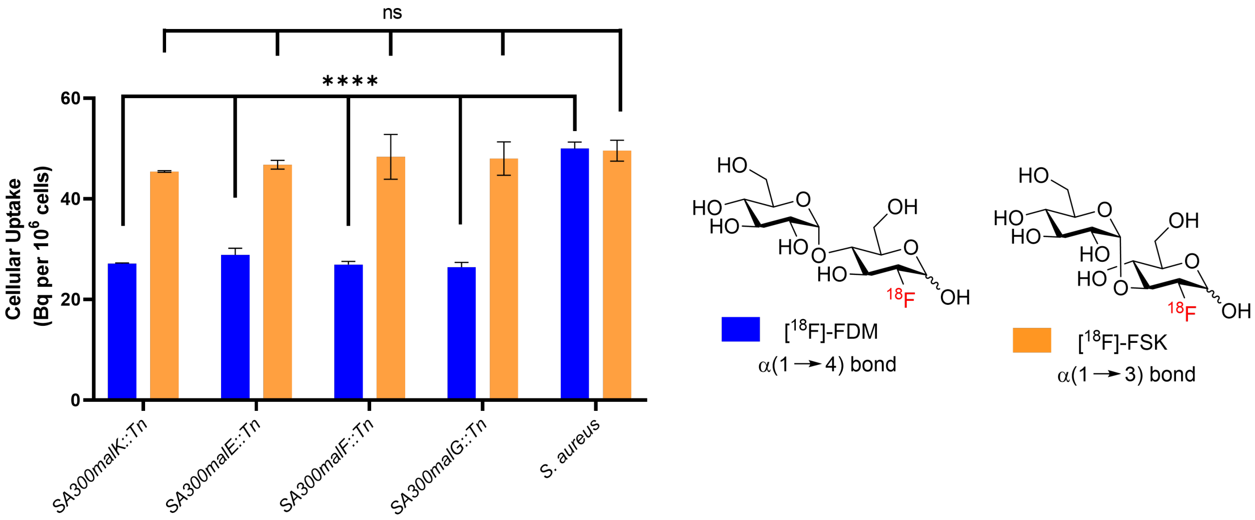

Figure S8. Uptake of [^{18}F]FDM and [^{18}F]FSK in S. aureus mutants in which the genes encoding the maltodextrin transporter have been disrupted. For [^{18}F]FDM, *Wild type vs mutant:* **** *P* value <0.0001 (unpaired t-test). For [^{18}F]FSK, *Wild type vs mutants*: ns (unpaired t-test).

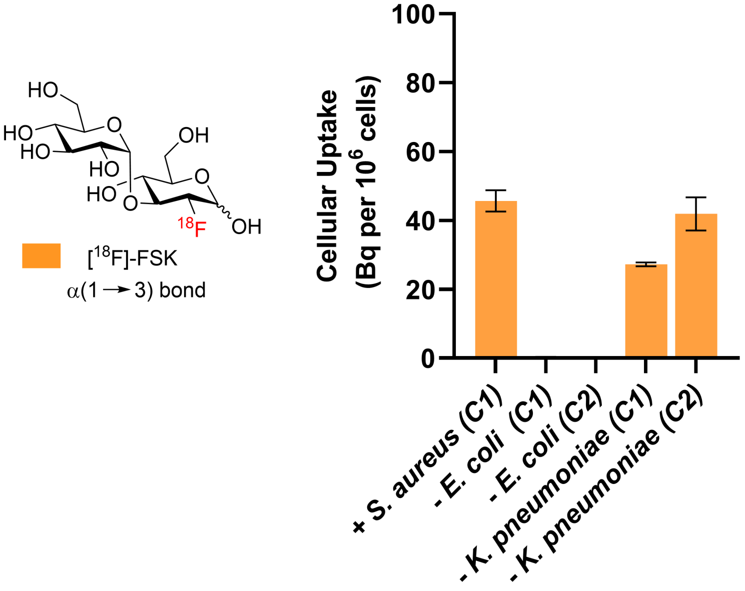

Figure S9. *In vitro* uptake of [^{18}F]FSK in clinical isolates of *S. aureus, E. coli and K. pneumoniae*.

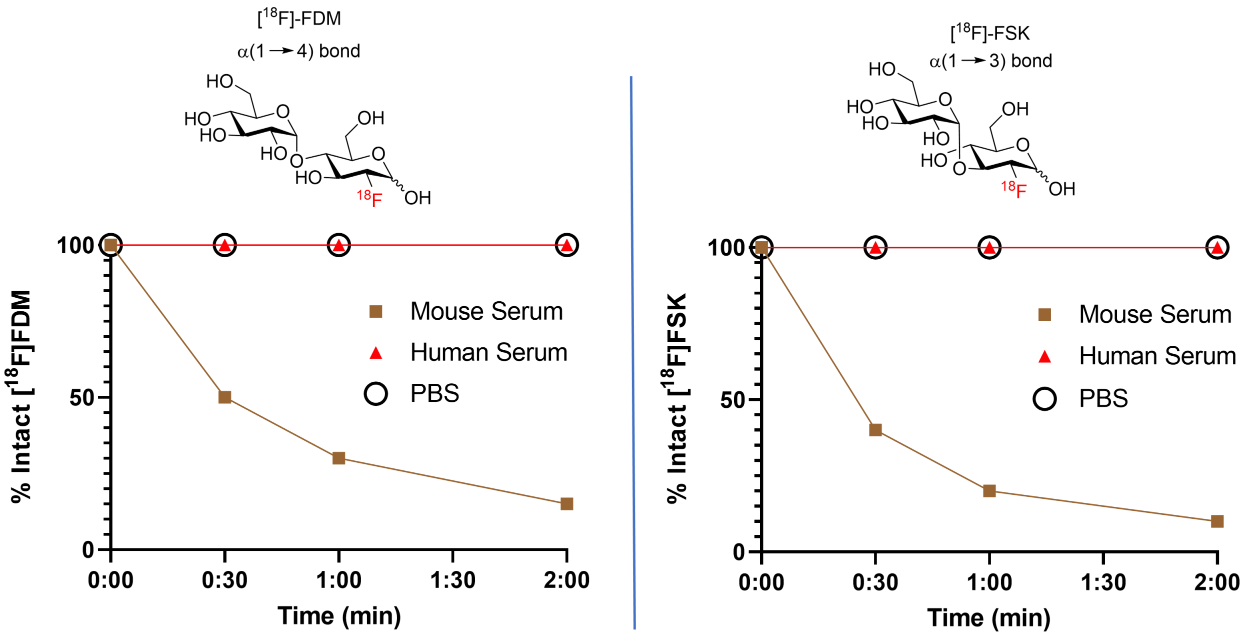

Figure S10. Stability of [^{18}F]FDM and [^{18}F]FSK in human and mouse serum.

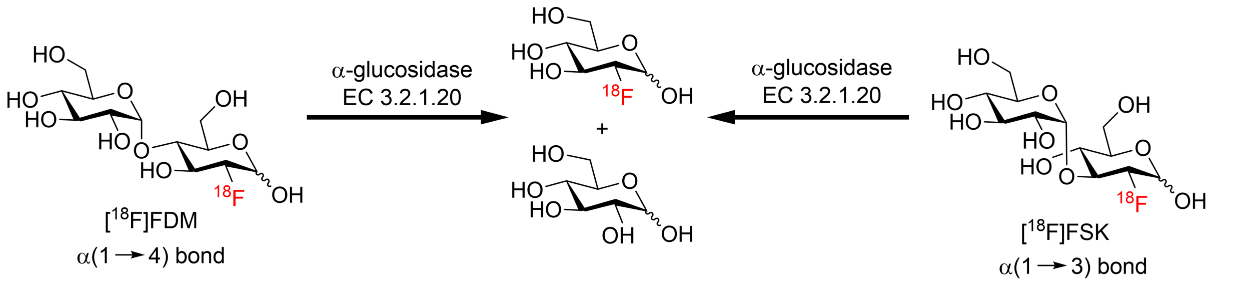

Figure S11. Schematic of α-glucosidase hydrolysis of [^{18}F]FDM and [^{18}F]FSK. In murine serum, α-glucosidase catalyzes the decomposition of [^{18}F]FDM and [^{18}F]FSK into [^{18}F]FDG and glucose.

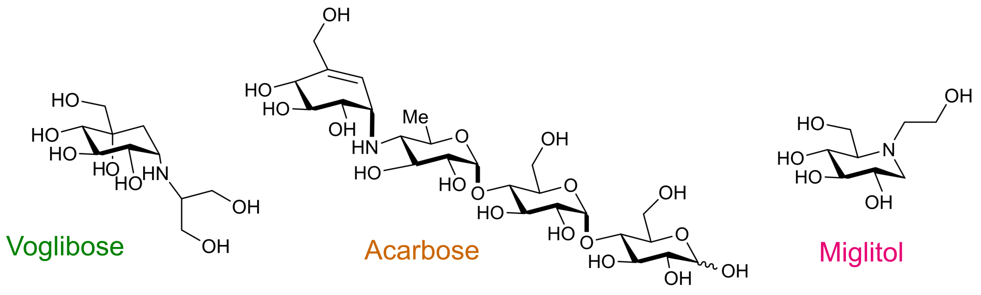

Figure S12. Inhibitors of α–glycosidase that have the potential for higher stability of [^{18}F]FDM and [^{18}F]FSK in mouse serum.

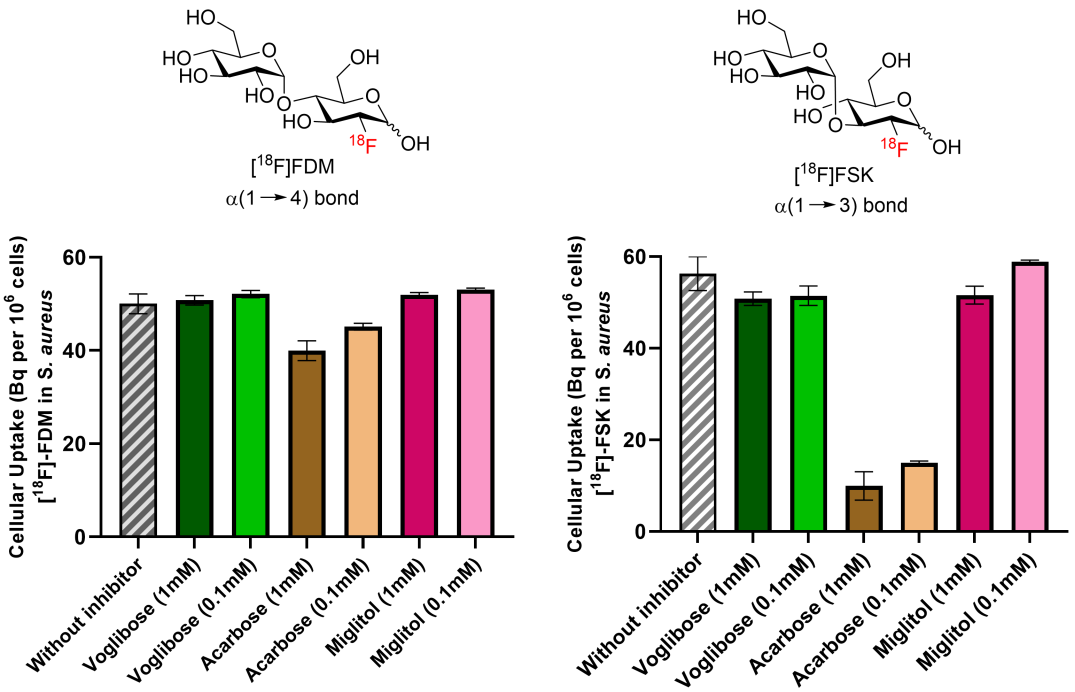

Figure S13. *In vitro S. aureus* uptake for [^{18}F]FDM and [^{18}F]FSK in the presence of the indicated α-glucosidase inhibitors.

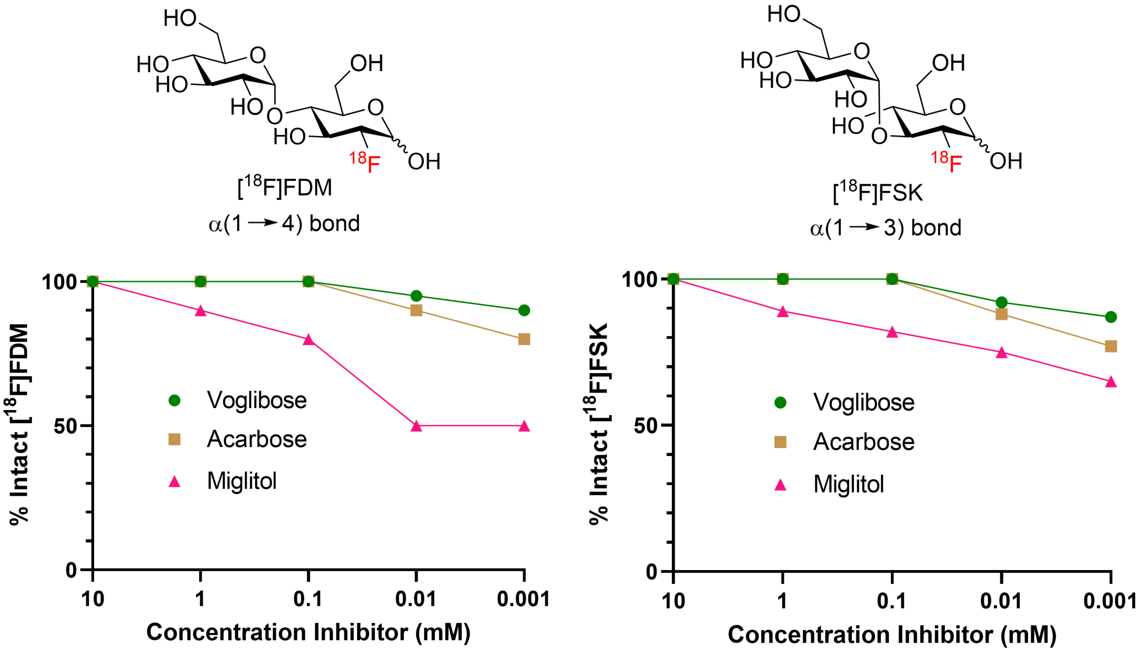

Figure S14. Dose response of [^{18}F]FDM and [^{18}F]FSK in mouse serum exposed to increasing concentrations of α-glucosidase inhibitors (0.001 to 10 mM).

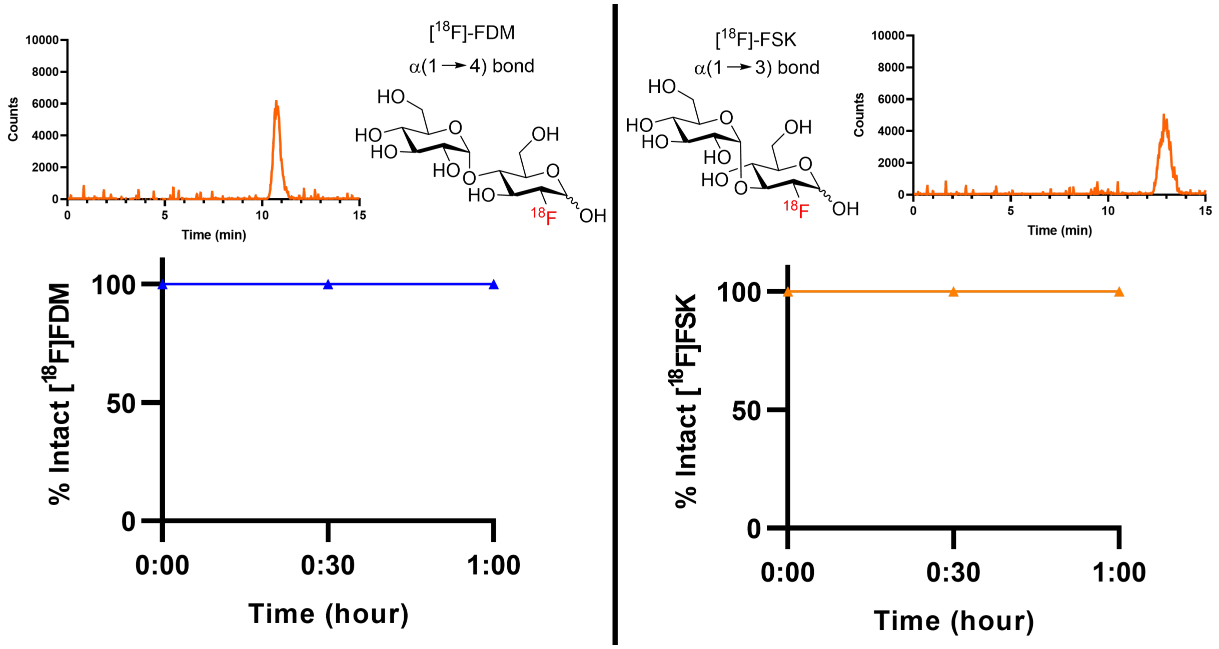

Figure S15. Stability of [^{18}F]FDM and [^{18}F]FSK in human liver microsomes (HLM). 100 μCi of [^{18}F]-tracer in 50 uL PBS was added to 200 μL pre-mixed and pre-incubated mixture of HLM (1mg/mL) and NADPH (2mM) in PBS. The final mixture was incubated at 37°C for 30 min and 1 hour. Samples of 20 μL were taken and added to 80 μL of cold MeCN (-20°C), which was shaken and centrifuged. The supernatant was injected on radio HPLC for analysis. The experimental procedure was adapted from (Ludwig et al. *Front. Pharmacol.* **2019**, *10*, 534).

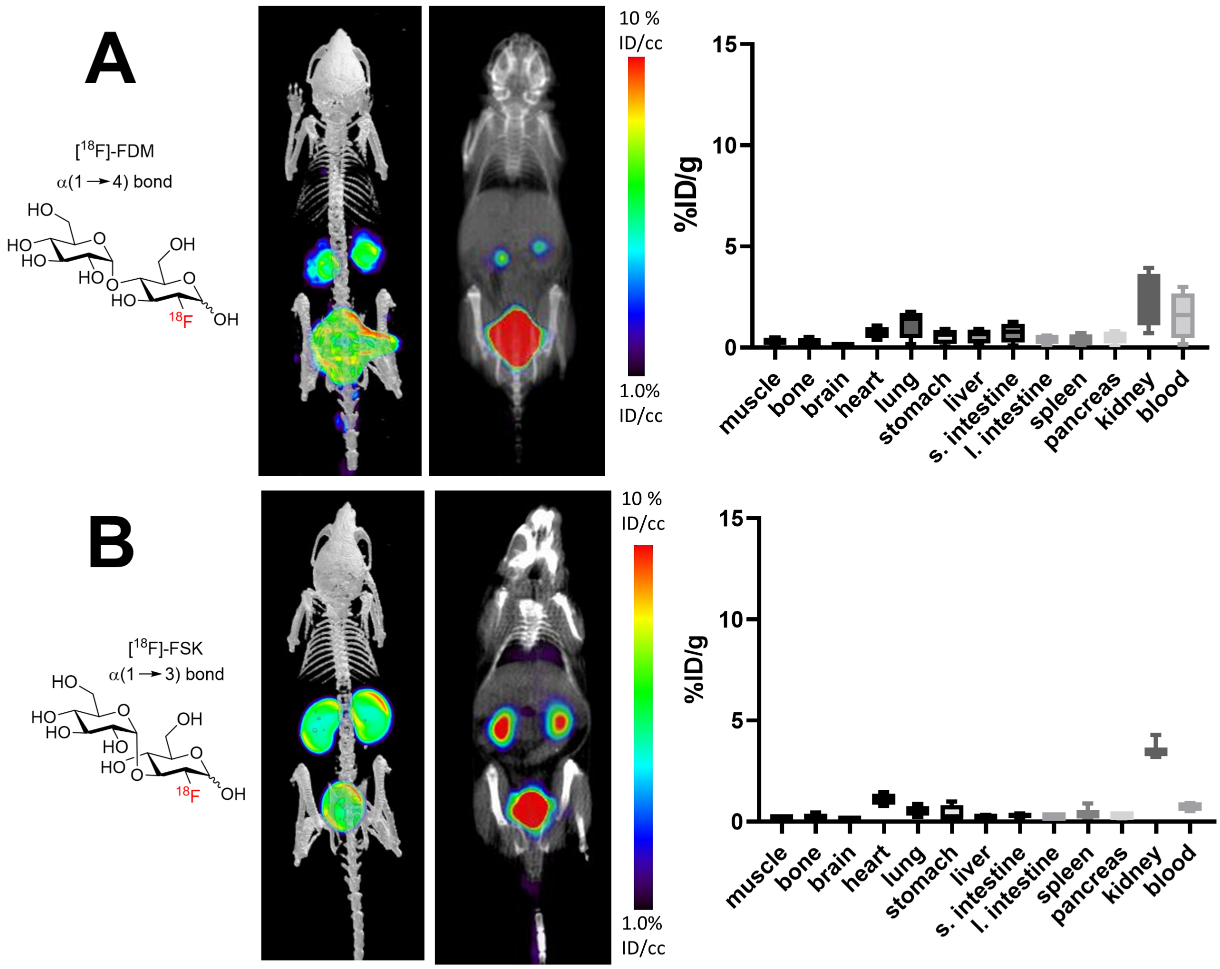

Figure S16. *In vivo* experiment: [^{18}F]FDM and [^{18}F]FSK in mouse, No bacteria, Inhibitor: Voglibose (1mg/ inj), Injection: ≈200uCi 18F-tracer, N = 5. Images were obtained 90 min after injection. *Ex vivo* data was obtained following tissue harvesting on a gamma counter.

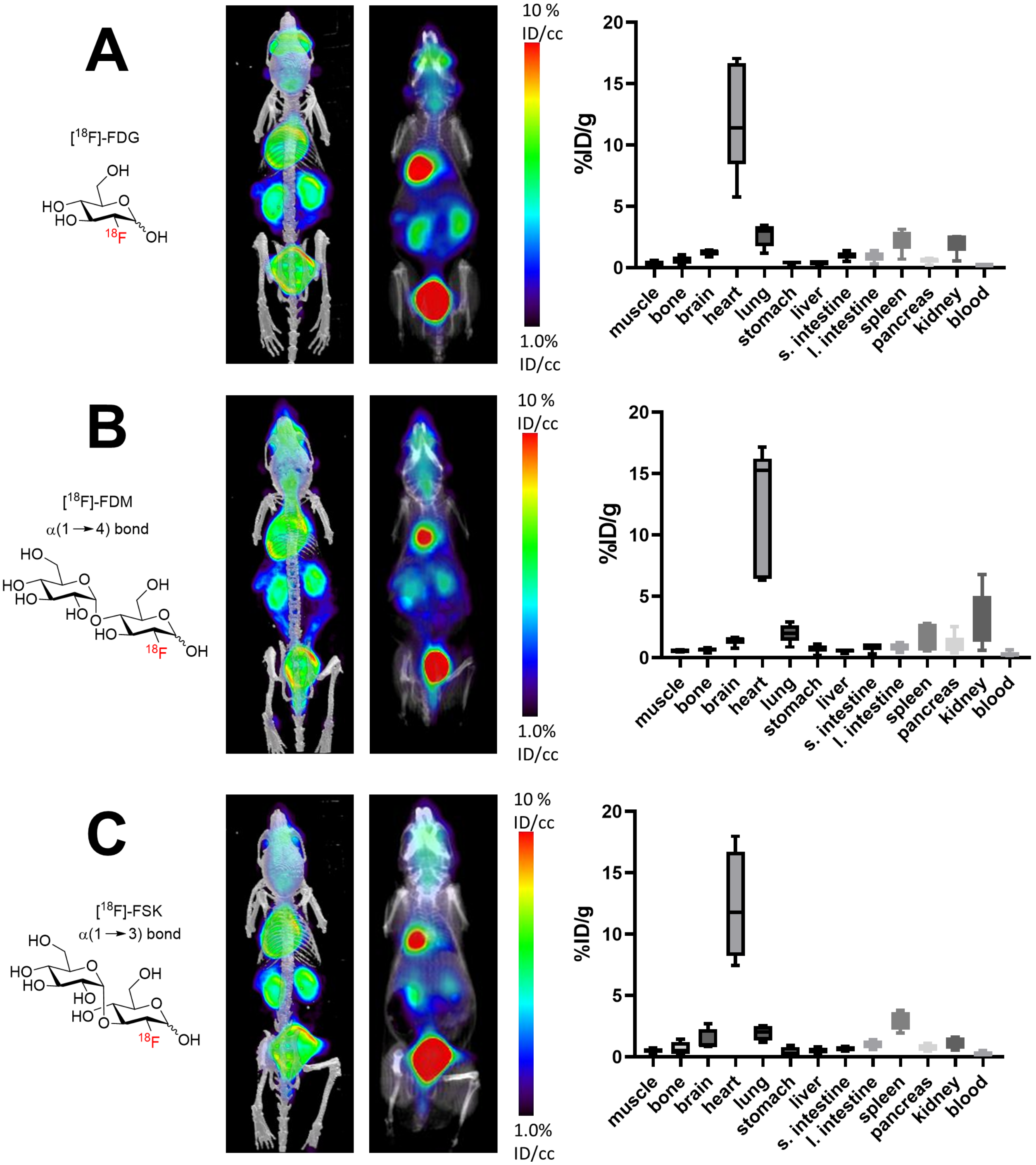

Figure S17. *In vivo* experiment: [^{18}F]FDG, [^{18}F]FDM and [^{18}F]FSK in mouse, No bacteria, No inhibitor, Injection: ≈200uCi 18F-tracer, N = 5. Images were obtained 90 min after injection. *Ex vivo* data was obtained following tissue harvesting on a gamma counter.

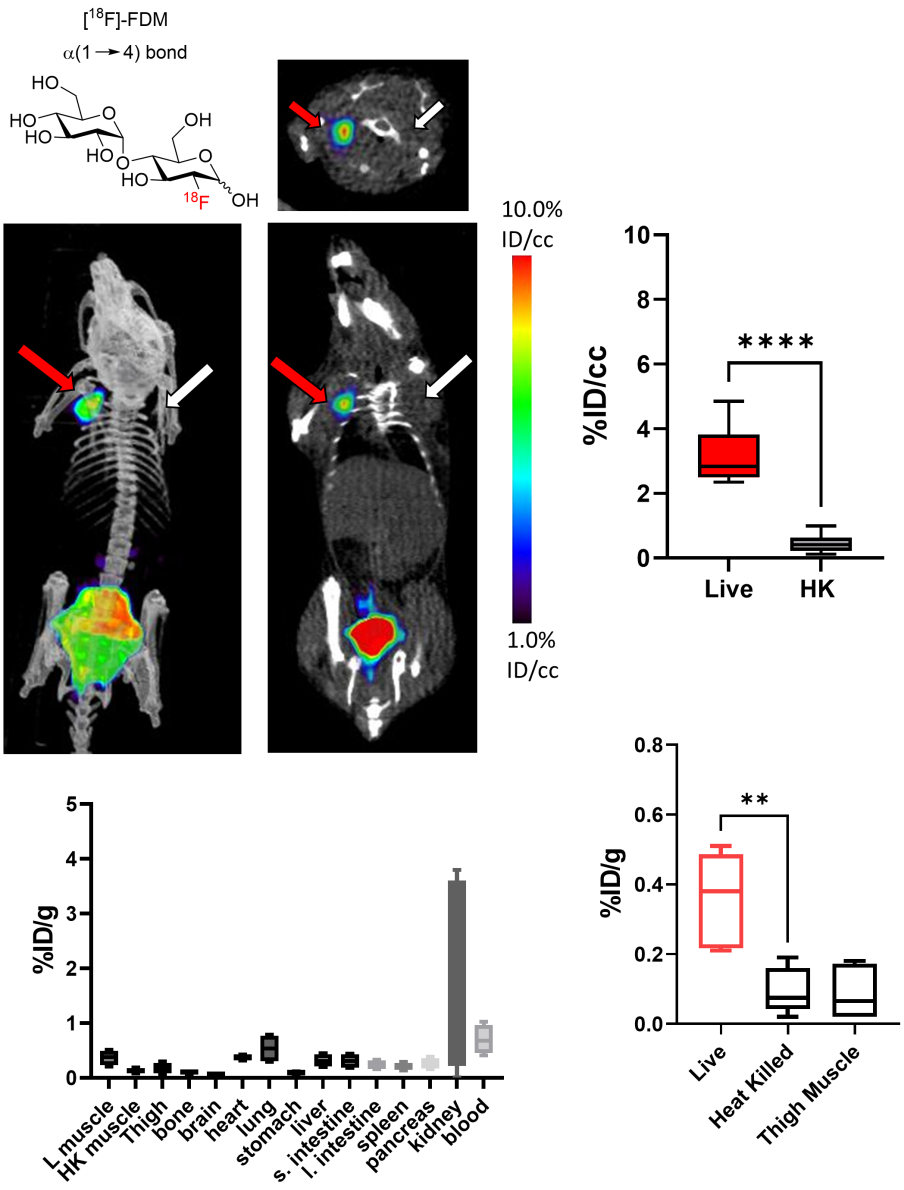

Figure S18. *In vivo* experiment: [^{18}F]FDM in mouse myositis model, *S. aureus* (MRSA 01), Inhibitor: Voglibose (1mg/ inj), Injection: ≈200uCi 18F-tracer, N = 6. The red arrows indicate the site of inoculation with live bacteria, while white arrows indicate the site of inoculation with heat-killed bacteria. Images were obtained 90 min after injection. *Ex vivo* data was obtained following tissue harvesting on a gamma counter. ROI analysis, *live vs HK*: 6.1-fold excess, **** *P* value <0.0001, *ex vivo* analysis, *live vs HK*: 3.9-fold excess, ** *P* value = 0.001 (unpaired t-test).

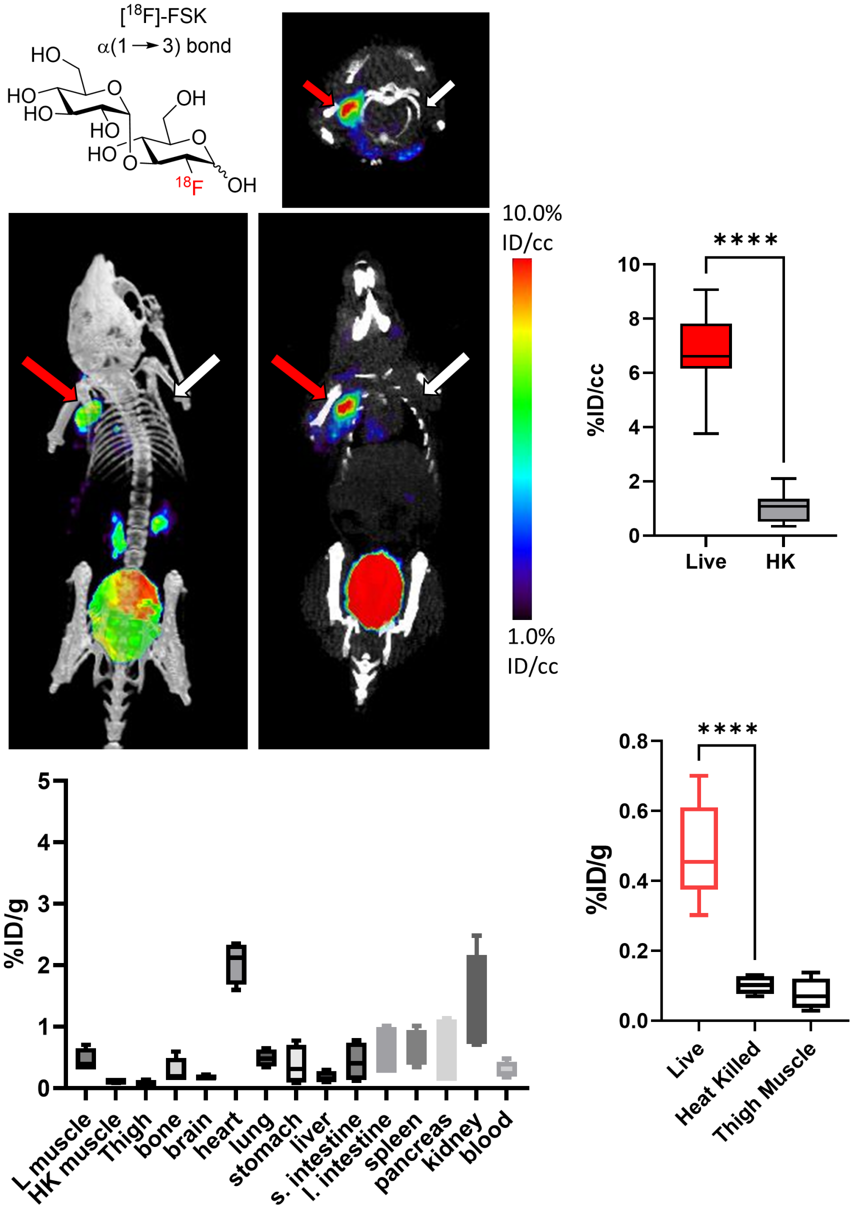

Figure S19. *In vivo* experiment: [^{18}F]FSK in mouse myositis model, *S. aureus* (MRSA 01), Inhibitor: Voglibose (1mg/ inj), Injection: ≈200uCi 18F-tracer, N = 6. The red arrows indicate the site of inoculation with live bacteria, while white arrows indicate the site of inoculation with heat-killed bacteria. Images were obtained 90 min after injection. *Ex vivo* data was obtained following tissue harvesting on a gamma counter. ROI analysis, *live vs HK*: 6.5-fold excess, **** *P* value <0.0001, *ex vivo* analysis, *live vs HK*: 4.7-fold excess, **** *P* value < 0.0001 (unpaired t-test).

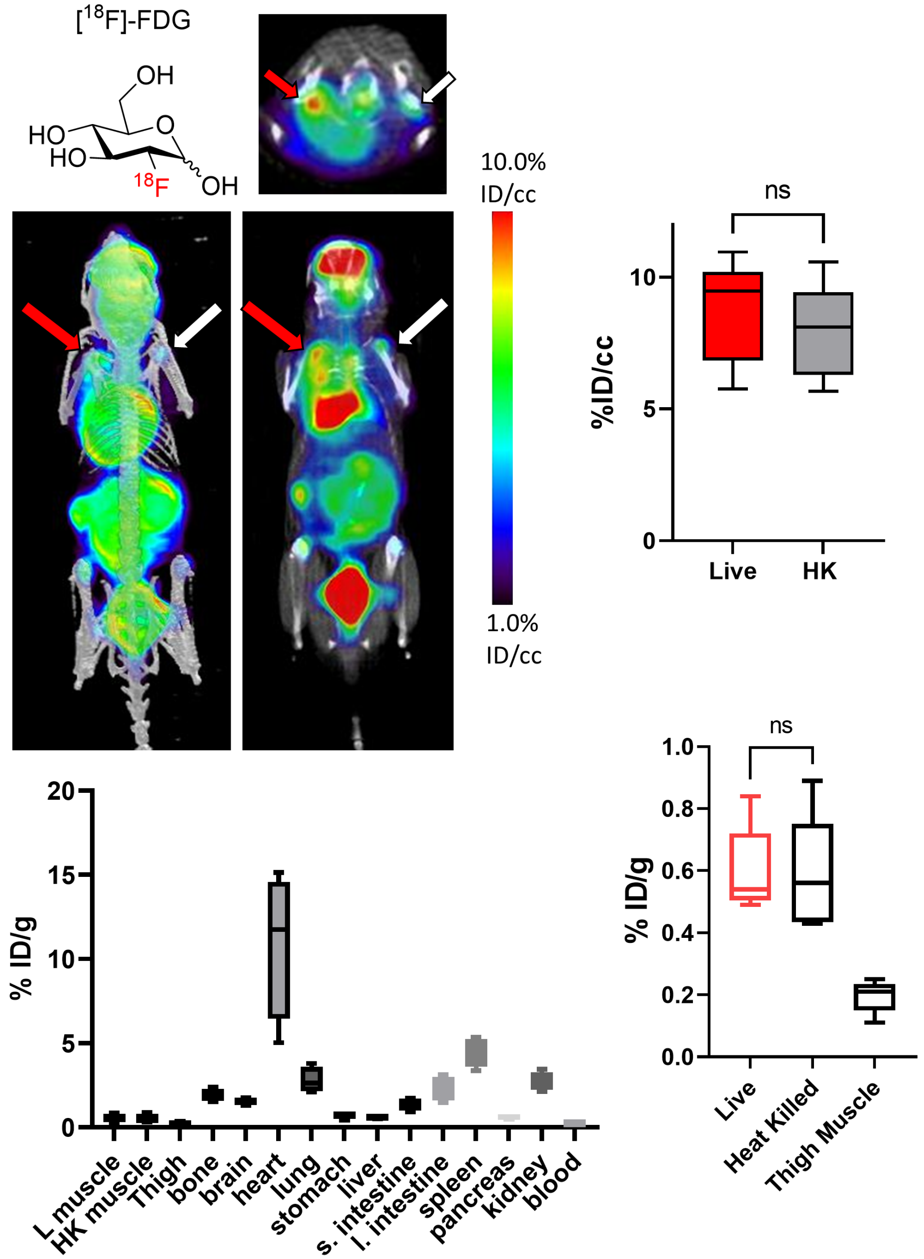

Figure S20. *In vivo* experiment: [^{18}F]FDG in mouse myositis model, *S. aureus* (MRSA 01), Inhibitor: Voglibose (1mg/ inj), Injection: ≈200uCi 18F-tracer, N = 5. The red arrows indicate the site of inoculation with live bacteria, while white arrows indicate the site of inoculation with heat-killed bacteria. Images were obtained 90 min after injection. *Ex vivo* data was obtained following tissue harvesting on a gamma counter. ROI analysis, *live vs HK*: 1.10-fold excess, ns, *ex vivo* analysis, *live vs HK*: 1.02-fold excess, ns (unpaired t-test).

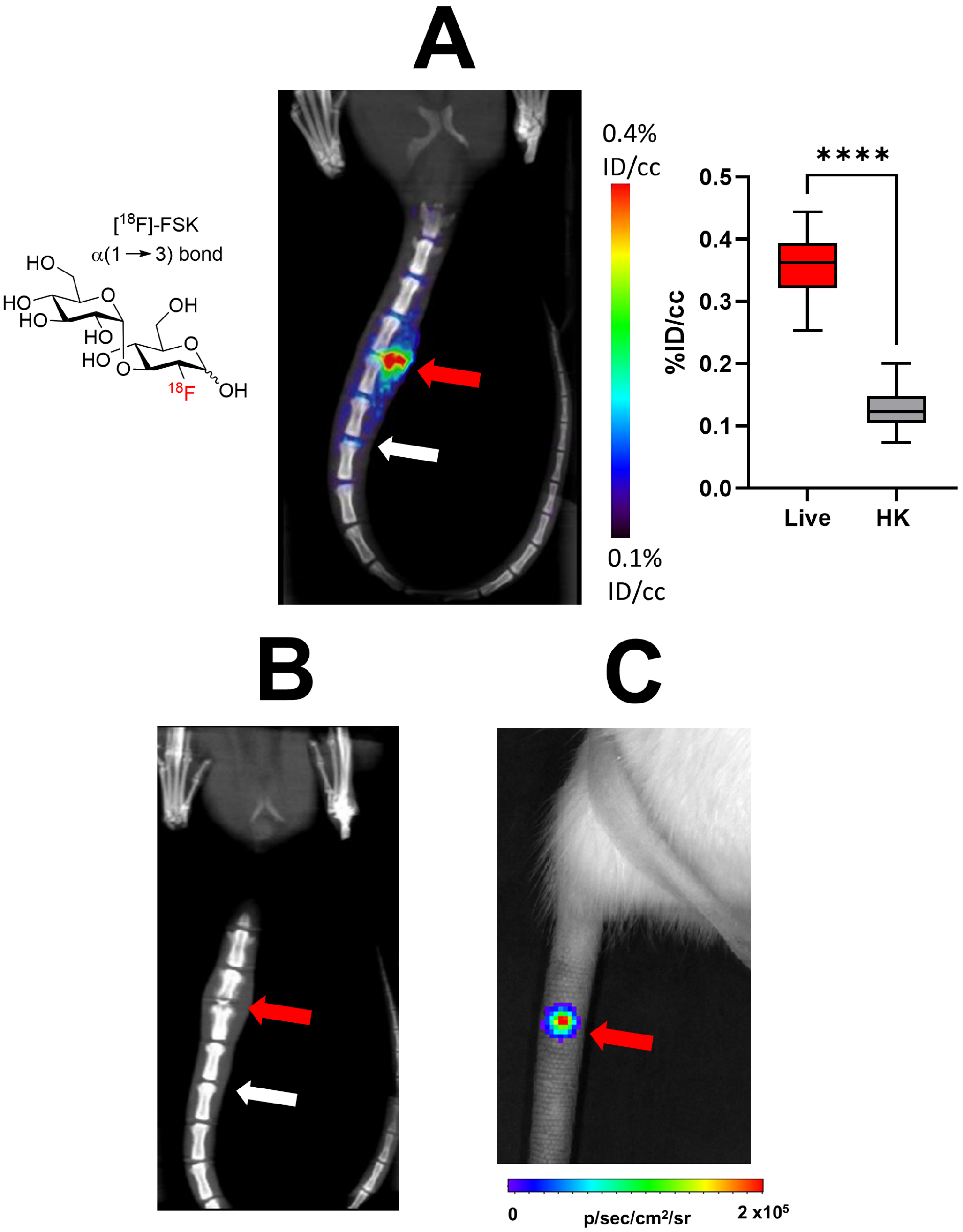

Figure S21 *In vivo* experiment: [^{18}F]FSK in rat vertebral discitis-osteomyelitis (VDO), *S. aureus* (Xen29), Day 4, inhibitor: Voglibose (5mg/ inj), Injection: ≈500uCi 18F-tracer, N = 5. The red arrows indicate the site of inoculation with live bacteria, while white arrows indicate the site of inoculation with heat-killed bacteria. (A) PET/CT imaging of *S. aureus* Xen29 vertebral discitis-osteomyelitis (VDO) in rat (*N* = 5) with [^{18}F]FSK. Images were obtained 90 min after injection. ROI analysis, *live vs HK*: 2.8-fold excess, **** *P* value <0.0001 (unpaired t-test). (B) Computed tomography study performed at 10 days highlights the similarity between rodent and human discitis osteomyelitis. (C) Optical tomography image of rat tail showing bioluminescent signal from *S. aureus* Xen29 inoculation.

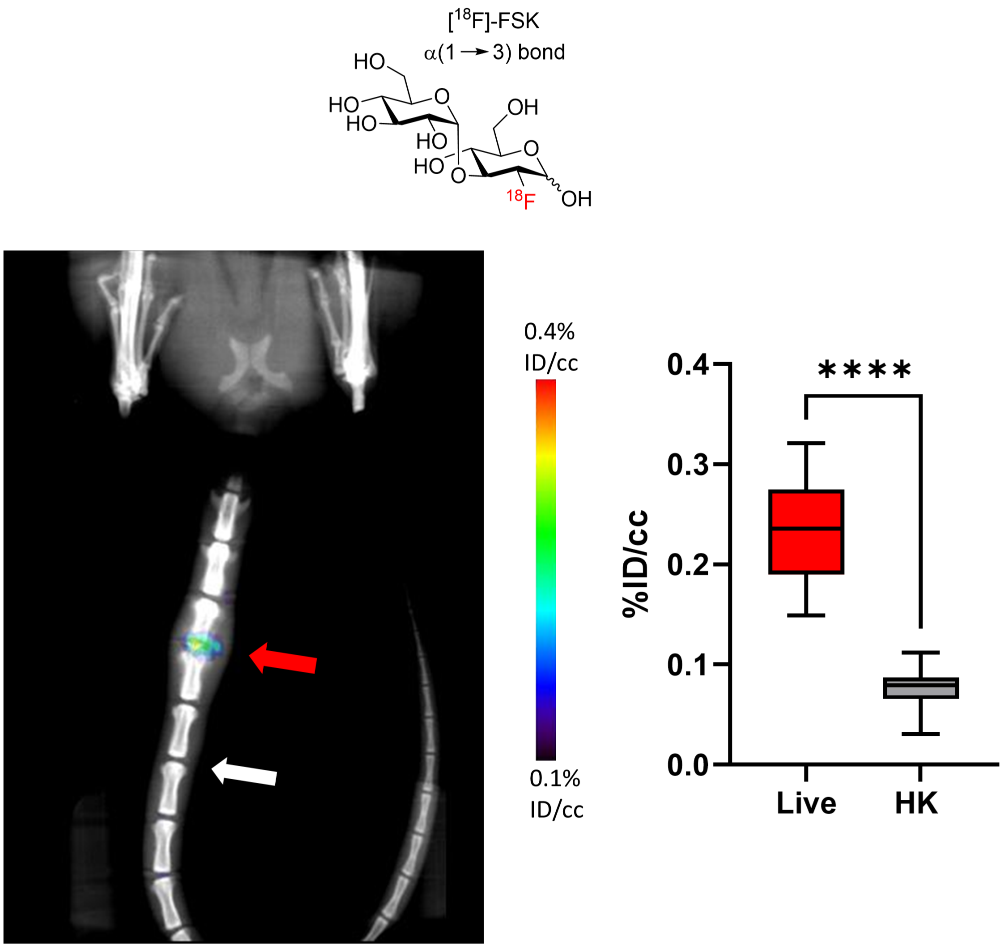

Figure S22. *In vivo* experiment: [^{18}F]FSK in rat vertebral discitis-osteomyelitis (VDO), *S. aureus* (Xen29), Day 10, inhibitor: Voglibose (5mg/ inj), Injection: ≈500uCi 18F-tracer, N = 3. The red arrows indicate the site of inoculation with live bacteria, while white arrows indicate the site of inoculation with heat-killed bacteria. Images were obtained 90 min after injection. ROI analysis, *live vs HK*: 3.1-fold excess, **** *P* value <0.0001 (unpaired t-test).

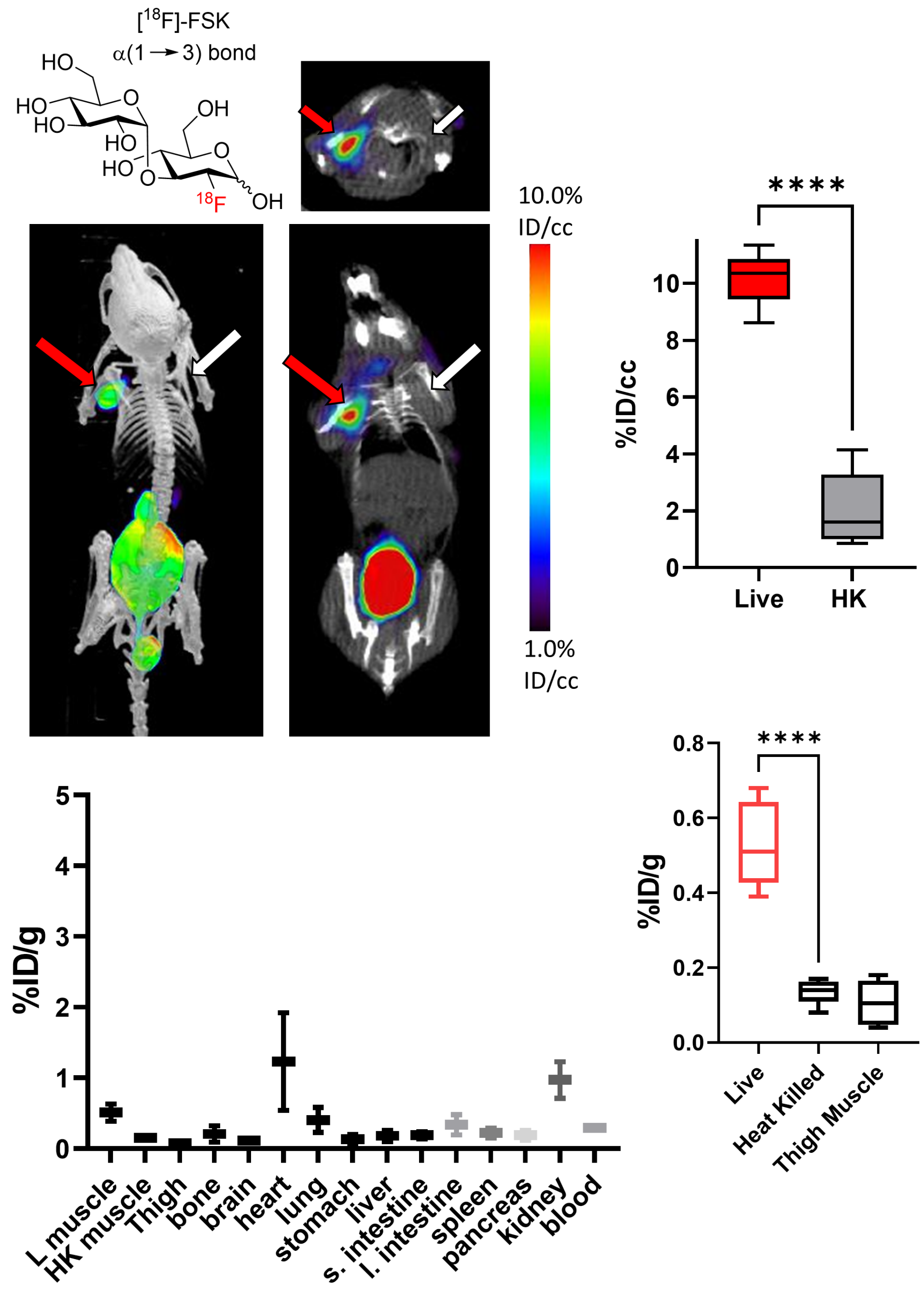

Figure S23. *In vivo* experiment: [^{18}F]FSK in mouse myositis model, *A. baumanni* (ATCC 19606), inhibitor: Voglibose (1mg/ inj), Injection: ≈200uCi 18F-tracer, N = 6. The red arrows indicate the site of inoculation with live bacteria, while white arrows indicate the site of inoculation with heat-killed bacteria. Images were obtained 90 min after injection. *Ex vivo* data was obtained following tissue harvesting on a gamma counter. ROI analysis, *live vs HK*: 4.9-fold excess, **** *P* value <0.0001, *ex vivo* analysis, *live vs HK*: 3.9 fold excess, **** *P* value < 0.0001 (unpaired t-test).

Table S1. Bacterial strains

The bacterial strains included in this study are listed in the table below.

**Strain**	**Phenotype or Genotype**	**Source or Reference**
S. aureus	Wild-type	ATCC 12600
S. aureus Xen29	ATCC 12600 expressing the *Photorhabdus luminescens luxABCDE* genes	Xenogen USA
S. aureus MRSA 1	MRSA Clinical isolate	University of Nebraska Medical Center
S. aureus MRSA 2	MRSA Clinical isolate	University of Nebraska Medical Center
S. aureus MRSA 3	MRSA Clinical isolate	University of Nebraska Medical Center
S. aureus MRSA 4	MRSA Clinical isolate	University of Nebraska Medical Center
S. aureus C1	MSSA Clinical isolate	University of Nebraska Medical Center
SA300malK::Tn	SAUSA300_0208	University of Nebraska Medical Center
SA300malE::Tn	SAUSA300_0209	University of Nebraska Medical Center
SA300malF::Tn	SAUSA300_0210	University of Nebraska Medical Center
SA300malG::Tn	SAUSA300_0211	University of Nebraska Medical Center
L. monocytogenes	Wild-type	ATCC 15313
S. epidermidis	Wild-type	ATCC 35984
K. pneumoniae	Wild-type	ATCC 13883
K. pneumoniae C1	Clinical isolate	University of California, San Francisco
K. pneumoniae C2	Clinical isolate	University of California, San Francisco
E. coli	Wild-type	ATCC 25922
E. coli C1	Clinical isolate	University of California, San Francisco
E. coli C2	Clinical isolate	University of California, San Francisco
P. aeruginosa PA01	Wild-type	ATCC 10154

A. baumannii	Wild-type	ATCC 19606
S. typhimurium	Wild-type	ATCC 29630
P. mirabilis	Wild-type	ATCC 29906
E. cloacae	Wild-type	ATCC 7256
E. faecalis	Wild-type	ATCC 19433

Synthetic Procedures

B.1. General:

All chemical reagents were purchased from commercial sources (Acros Organics, Alfa Aesar, AK Scientific & Sigma-Aldrich) and used without further purification unless otherwise stated. All separatory cartridges were purchased from Waters. Reactions were monitored by thin layer chromatography (TLC) on precoated (250 μm) silica gel 60 F254 aluminum sheets and visualized under a UV-254 lamp followed by staining with potassium permanganate. Flash chromatography was performed on silica gel (60A pore size). ^{1}H, ^{13}C, ^{31}P and ^{19}F NMR spectra were obtained on a Bruker Avance III HD 400 mHz instrument at the UCSF Nuclear Magnetic Resonance Laboratory and data were processed using MestReNova. Abbreviations are as follows: s (singlet), d (doublet), t (triplet), q (quartet), m (multiplet). High resolution mass spectra (HRMS) services were provided by University of California, Berkeley Spectrometry Facility. The ^{18}F labeled compounds were characterized by developing the compounds in different solvent systems on silica gel TLC plates on glass followed by imaging on a radio TLC scanner (Bioscan AR2000). Analytical HPLC was performed using a Waters pump equipped with a manual Rheodyne injector (1 mL loop), a refracted index (RI) detector and a RAD detector. The stationary phase was YMC-Pack Polyamine II column and the mobile phase of 73:27 acetonitrile/H_2O at a flowrate of 1 mL/min. For semi prep HPLC, a YMC-Pack Polyamine II stationary phase was used with a mobile phase of 73:27 acetonitrile/H_2O at a flowrate of 4 mL/min, The radioactivity of the bacterial pallets and filtrate were counted on a γ counter (Hidex Automatic Gamma Counter).

B.2. Synthesis of precursor: β-D-glucose-1-phosphate (βGlc1-P)

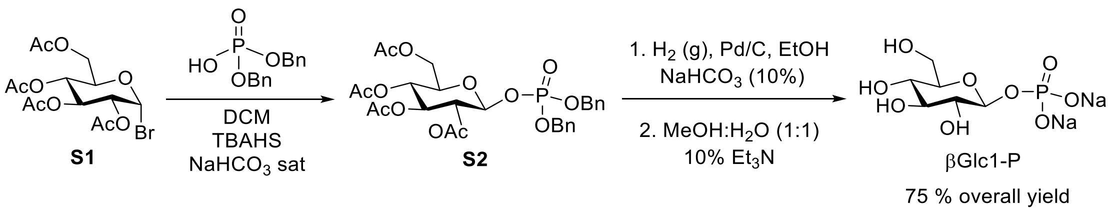

Dibenzyl(2,3,4,6-tetra-*O*-acetyl-β-D-glucopyranosyl) phosphate (**S2**).

In a 250 mL Erlenmeyer flask, dibenzyl phosphate (6.8 g, 24.4 mmol) and tetrabutylammonium hydrogen sulfate (4.1 g, 12.2 mmol) were added and mixed with 120 mL of saturated $NaHCO_3$ aqueous solution. After stirring at room temperature for 10 min, the mixture was added to a 500 mL round bottom flask containing acetobromo-α-D-glucose **S1** (5.0 g, 12.2

mmol) in DCM (120 mL). The biphasic reaction mixture was capped then stirred vigorously at room temperature for 72 hrs. Over the reaction time, the pH of the aqueous phase was maintained at pH 8-9 via addition of saturated $NaHCO_3$ solution. The organic phase was extracted using ethyl acetate, before being washed with saturated $NaHCO_3$, water, and brine. The organic phase was then dried with sodium sulfate, filtered, and evaporated under reduced pressure. The residue was purified via silica flash chromatography using 1% *t*-butanol in DCM (with a few drops of Et_3N) as eluant to give pure phosphate **S2** (5.2 g, 71% yield).

^{1}H NMR (400 MHz, $CDCl_3$) δ 7.46 – 7.30 (m, 10H), 5.38 (t, *J* = 7.6 Hz, 1H), 5.23 (d, *J* = 9.4 Hz, 1H), 5.16 (d, *J* = 7.8 Hz, 1H), 5.14 (d, *J* = 6.3 Hz, 1H), 5.11 (d, *J* = 7.4 Hz, 2H), 5.04 (d, *J* = 7.1 Hz, 2H), 4.26 (dd, *J* = 12.5, 4.8 Hz, 1H), 4.14 (dd, *J* = 12.4, 2.1 Hz, 1H), 3.83 (ddd, *J* = 10.0, 4.8, 2.2 Hz, 1H), 2.06 (s, 3H), 2.03 (s, 6H), 1.92 (s, 3H).

^{31}P NMR (162 MHz, $CDCl_3$) δ -3.20 (dd, *J* = 14.5, 7.3 Hz). The NMR values matched the reported literature.[1]

<u>β-D-Glucose-1-phosphate (βGlc1-P).</u>

In a 100 mL round bottom flask, phosphate **S2** (0.5 mg, 0.82 mmol) was hydrogenated (14.7 psi) over 5% Pd/C (94.3 mg), 14 mL of EtOH and 9 mL of 10% $NaHCO_3$ for 16 hrs at room temperature. The mixture was then filtered then concentrated. The residue was transferred to a 25 mL round bottom flask, to which was added Et_3N (0.4 mL) and a 1:1 mixture of MeOH/H_2O (5.5 mL). The mixture was stirred for 16 hrs before being concentrated then diluted with H_2O. The pH was lowered using Dowex 50W-X8 [H^+] resin until pH 7, before residue was filtered then lyophilized to obtain β-D-glucose-1-phosphate (βGlc1-P), (201 mg, 80 % yield).

^{1}H NMR (400 MHz, D_2O) δ 4.90 (t, *J* = 7.7 Hz, 1H), 3.91 (dd, *J* = 12.3, 2.1 Hz, 1H), 3.68 (dd, *J* = 12.3, 6.9 Hz, 1H), 3.56 – 3.47 (m, 2H), 3.34 (t, 1H), 3.31 (t, 1H).

^{31}P NMR (162 MHz, D_2O) δ 2.29 (d, *J* = 7.7 Hz). The NMR values matched the reported literature.[2]

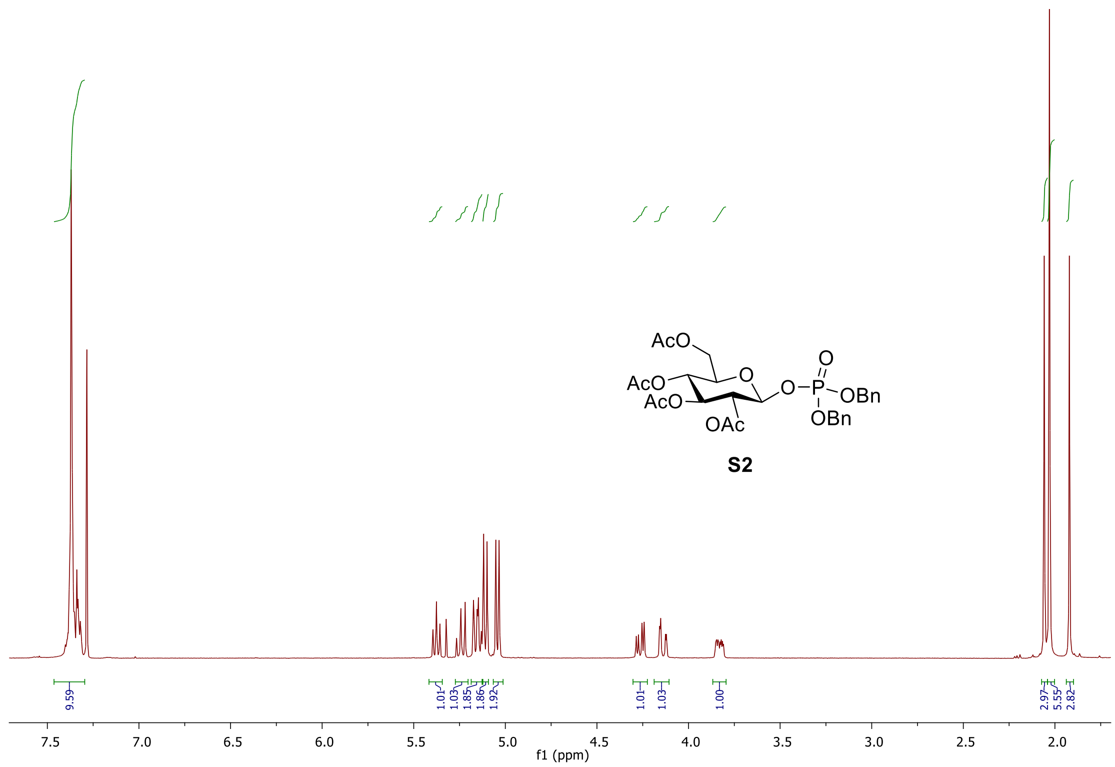

Figure B.2.1. ^{1}H NMR of (**S2**) in $CDCl_3$.

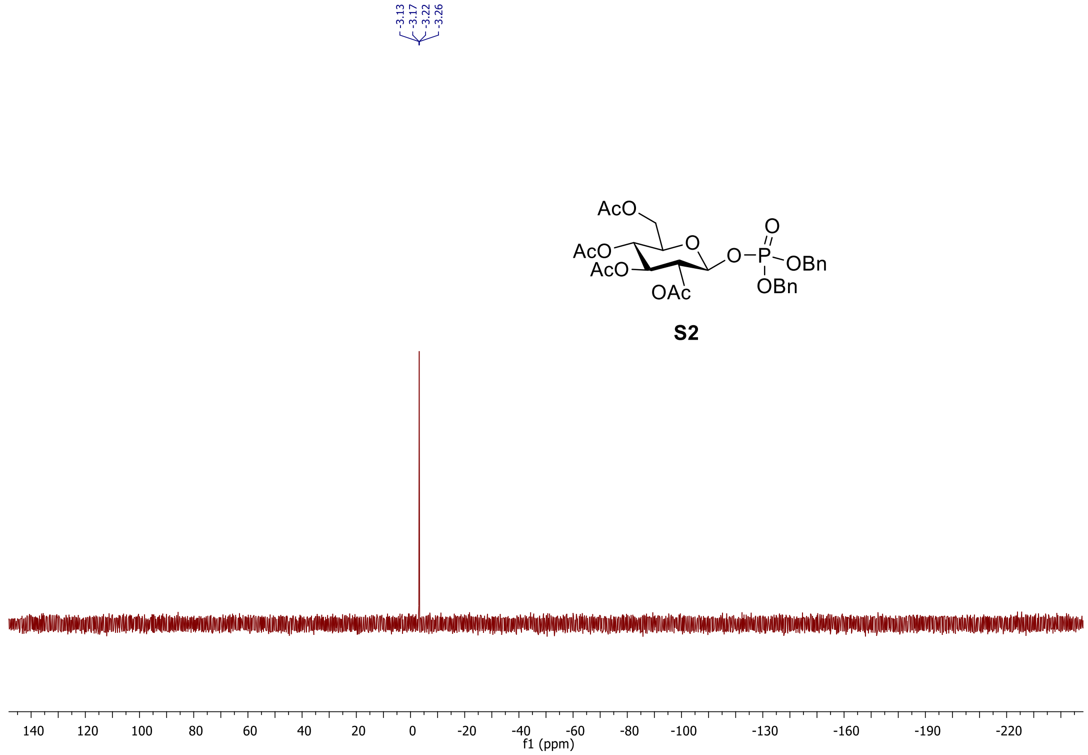

Figure B.2.2. ^{31}P NMR of (**S2**) in $CDCl_3$.

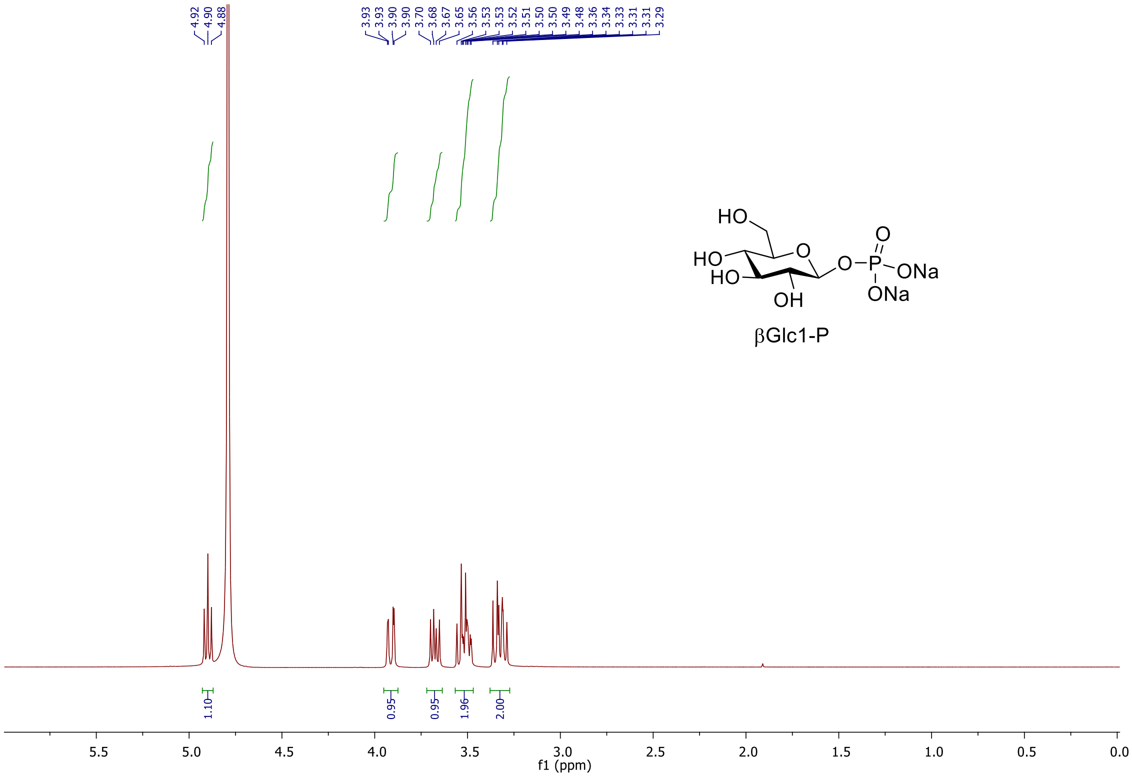

Figure B.2.3. ^{1}H NMR of β-D-glucose-1-phosphate in D_2O.

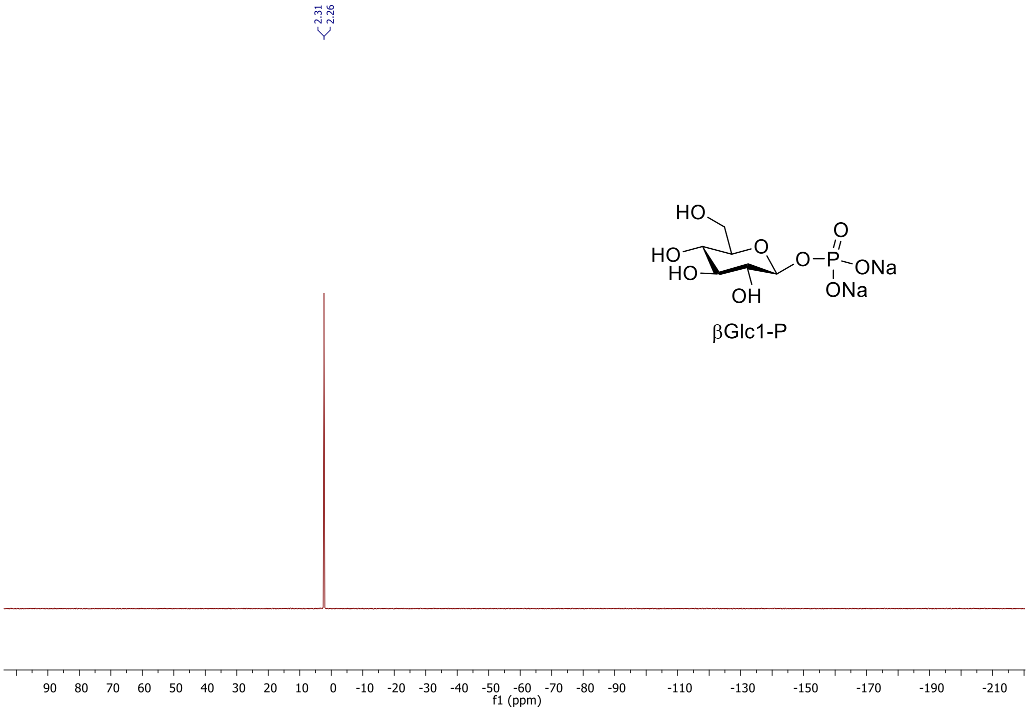

Figure B.2.4. ^{31}P NMR of β-D-glucose-1-phosphate in D_2O.

B.3. Synthesis of ^{19}F Standards:

B.3.1. 2-deoxy-2-[19F]fluoro-maltose and 2-deoxy-2-[19F]fluoro-sakebiose

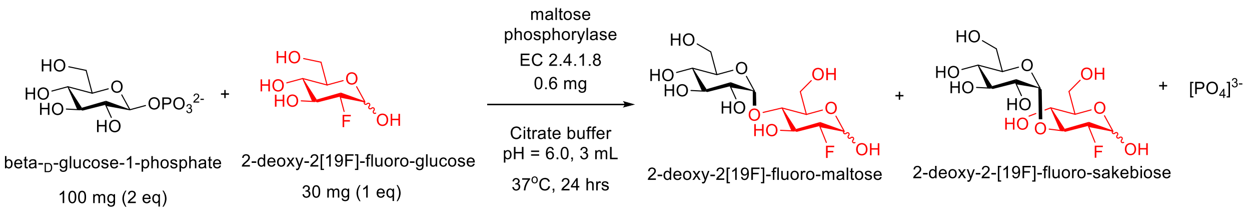

β-D-glucose-1-phosphate (βGlc1-P) (100 mg, 0.32 mmol), 2-deoxy-2[^{19}F]-fluoro-glucose (30 mg, 0.16 mmol) were added to a 10 mL round bottom flask containing maltose phosphorylase (EC 2.4.1.8, Sigma Aldrich), (0.6 mg, 6 units) in 3 mL of aqueous citrate buffer solution (pH = 6.0). The mixture was stirred for 24 hrs at 37°C. The residue was diluted with MeCN then purified via semi prep HPLC (YMC-Pack Polyamine II, 250 X 10 mm) using 70% MeCN/30 % H_2O to yield 2-deoxy-2[^{19}F]-fluoro-maltose (22 mg, 41% yield) and 2-deoxy-2-[^{19}F]-fluoro-sakebiose (8 mg, 14% yield).

Semi-Prep HPLC Purification

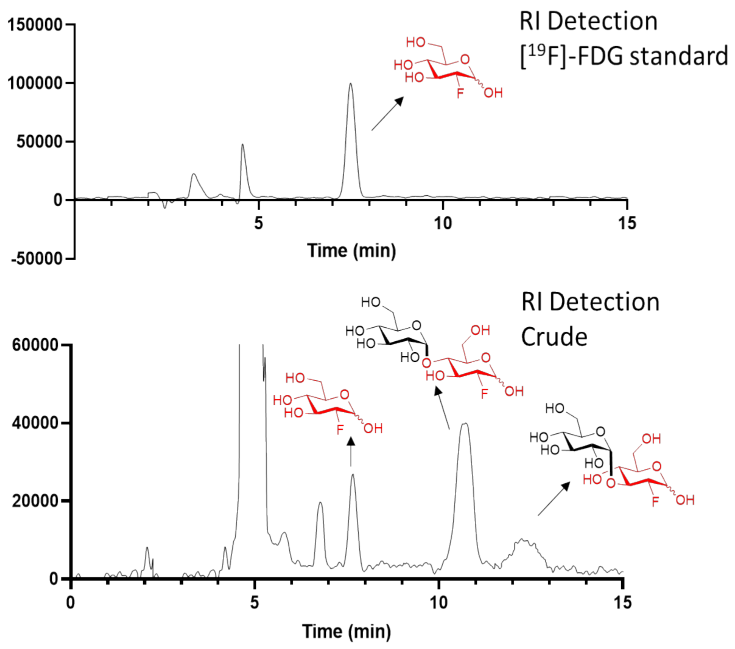

Figure B.3.1. Chromatograms for semi-prep HPLC purification of 2-deoxy-2[^{19}F]-fluoro-maltose and 2-deoxy-2-[^{19}F]-fluoro-sakebiose.

B.3.2. 2-deoxy-2-[^{19}F]fluoro-cellobiose

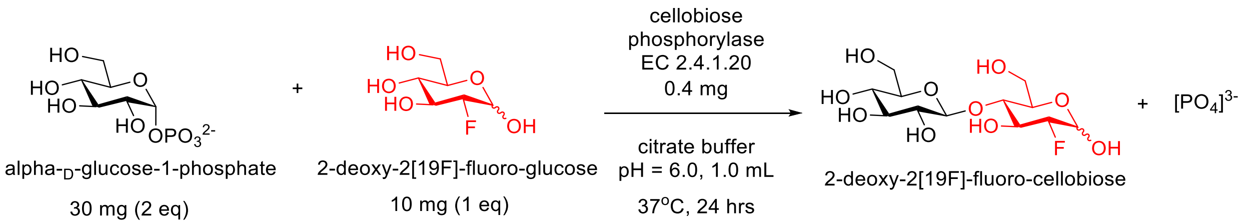

α-D-glucose-1-phosphate (αGlc1-P) (30 mg, 0.1 mmol), 2-deoxy-2[^{19}F]-fluoro-glucose (10 mg, 0.05 mmol) were added to a 5 mL round bottom flask containing cellobiose phosphorylase (EC 2.4.1.20), (0.4 mg, 6 units) in 1 mL of aqueous citrate buffer solution (pH = 6.0). The mixture was stirred for 24 hrs at 37°C. The residue was diluted with MeCN then purified via semi prep HPLC (YMC-Pack Polyamine II, 250 X 10 mm) using 70% MeCN/30 % H_2O to yield 2-deoxy-2[^{19}F]-fluoro-cellobiose (10 mg, 59% yield).

Semi-Prep HPLC Purification

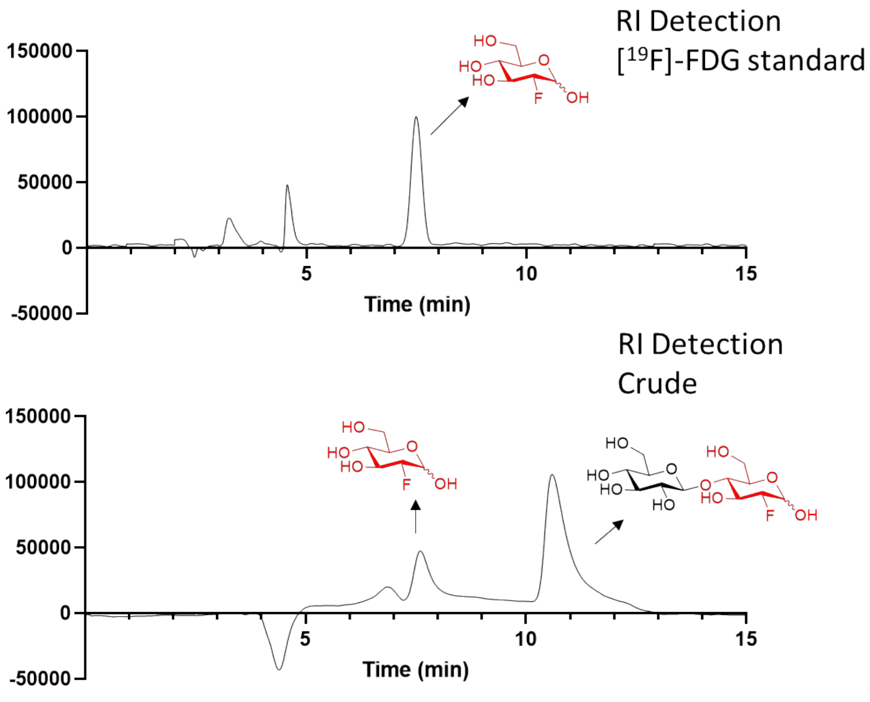

Figure B.3.2 Chromatograms for semi-prep HPLC purification of 2-deoxy-2[^{19}F]-fluoro-cellobiose.

B.3.3. 2-deoxy-2-[^{19}F]fluoro-trehalose

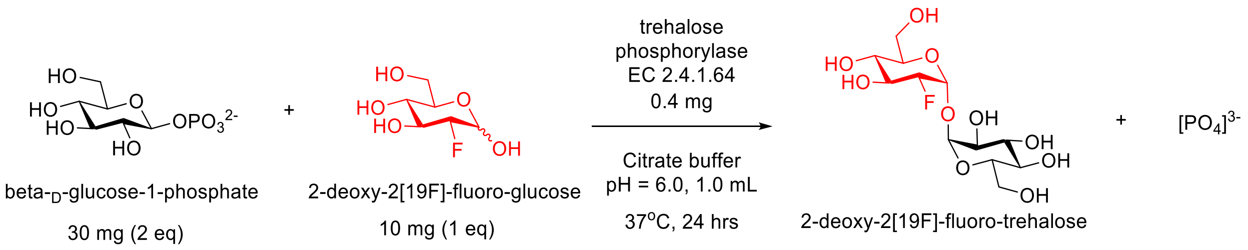

β-D-glucose-1-phosphate (βGlc1-P) (30 mg, 0.1 mmol), 2-deoxy-2[^{19}F]-fluoro-glucose (10 mg, 0.05 mmol) were added to a 5 mL round bottom flask containing trehalose phosphorylase (EC 2.4.1.64), (0.4 mg, 6 units) in 1 mL of aqueous citrate buffer solution (pH = 6.0). The mixture was stirred for 24 hrs at 37°C. The residue was diluted with MeCN then purified via semi prep HPLC (YMC-Pack Polyamine II, 250 X 10 mm) using 70% MeCN/30 % H_2O to yield 2-deoxy-2[^{19}F]-fluoro-trehalose (8 mg, 47% yield).

Semi-Prep HPLC Purification

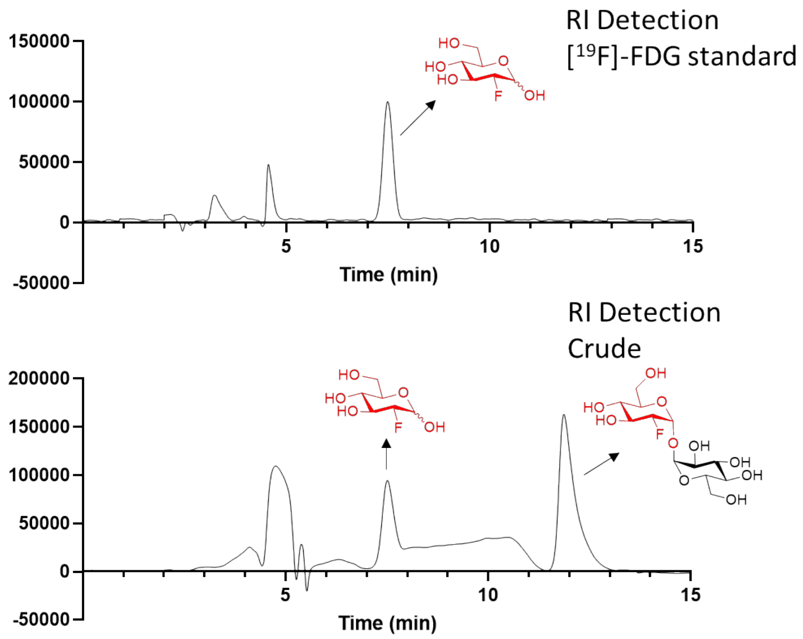

Figure B.3.3. Chromatograms for semi-prep HPLC purification of 2-deoxy-2[^{19}F]-fluoro-trehalose.

B.3.4. 2-deoxy-2-[19F]fluoro-laminaribiose

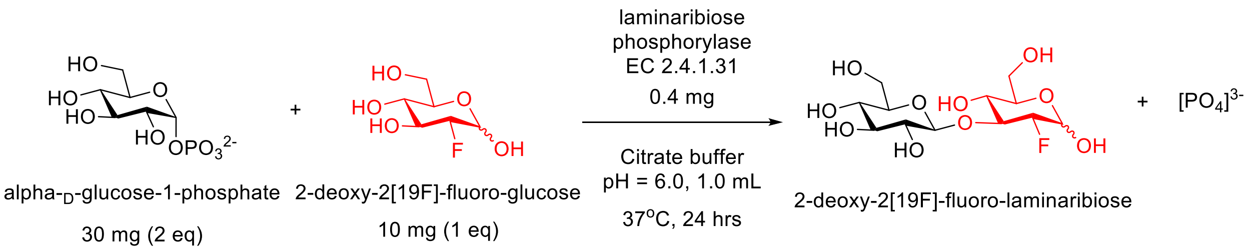

α-D-glucose-1-phosphate (αGlc1-P) (30 mg, 0.1 mmol), 2-deoxy-2[19F]-fluoro-glucose (10 mg, 0.05 mmol) were added to a 5 mL round bottom flask containing laminaribiose phosphorylase (EC 2.4.1.31), (0.4 mg, 6 units) in 1 mL of aqueous citrate buffer solution (pH = 6.0). The mixture was stirred for 24 hrs at 37°C. The residue was diluted with MeCN then purified via semi prep HPLC (YMC-Pack Polyamine II, 250 X 10 mm) using 70% MeCN/30 % H_2O to yield 2-deoxy-2[19F]-fluoro-laminaribiose (5 mg, 29% yield).

Semi-Prep HPLC Purification:

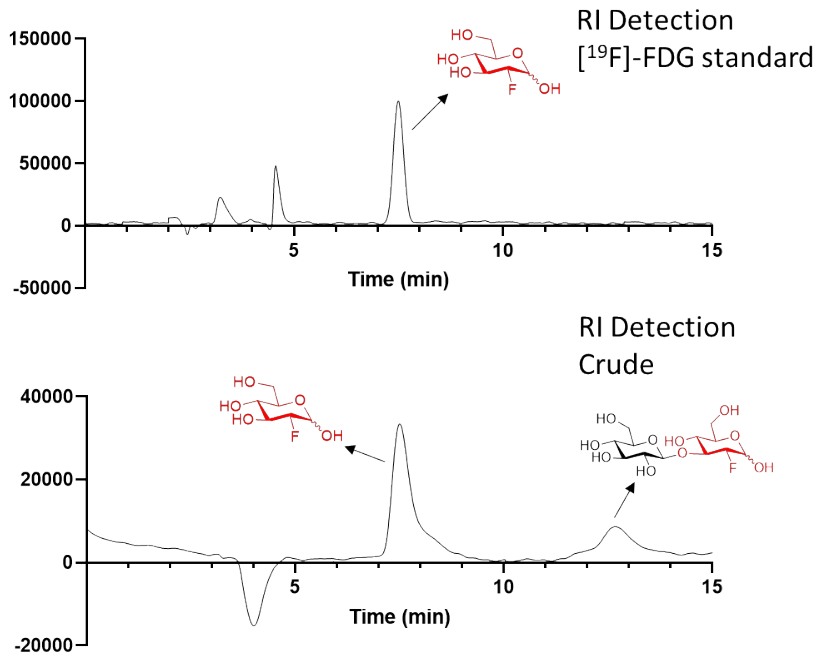

Figure B.3.4. Chromatograms for semi-prep HPLC purification of 2-deoxy-2[19F]-fluoro-laminaribiose.

NMR and HRMS analysis:

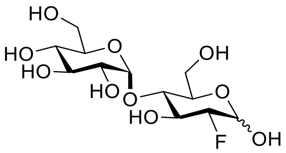

2-deoxy-2[^{19}F]-fluoro-maltose

^{1}H NMR (400 MHz, D_2O) δ 5.39 – 5.30 (m, 3H), 4.83 (dd, *J* = 7.7, 2.3 Hz, 1H), 4.36 (ddd, *J* = 49.2, 9.6, 3.9 Hz, 1H), 4.20 – 3.96 (m, 3H), 3.89 (ddd, *J* = 10.0, 4.4, 2.4 Hz, 1H), 3.86 – 3.78 (m, 2H), 3.77 – 3.73 (m, 2H), 3.72 – 3.53 (m, 11H), 3.49 (ddd, *J* = 9.9, 3.9, 0.8 Hz, 2H), 3.34 (td, *J* = 9.5, 1.4 Hz, 2H).

^{13}C NMR (100 MHz, D_2O) δ 99.57 (C1'α), 99.41 (C1'β), 93.39 (d, *J* = 23.1 Hz, C1β), 92.67 (d, *J* = 183.6 Hz, C2β), 89.95 (d, *J* = 186.0 Hz, C2α), 89.43 (d, *J* = 21.3 Hz, C1α), 75.96 (d, *J* = 14.6 Hz, C4α), 75.88 (d, *J* = 14.9 Hz, C4β), 74.58 (d, *J* = 6.5 Hz, C5β), 74.49 (d, *J* = 24.2 Hz, C3β), 72.77 (C5'α), 72.74 (C5'β), 72.67 (C3'α), 72.66 (C3'β), 71.66 (C2'α), 71.57 (d, *J* = 18.2 Hz, C3α), 71.56 (C2'β), 69.71(C5α), 69.26 (C4'α, C4'β), 60.49 - 60.31 (C6α, C6'α, C6β, C6'β).

^{19}F NMR (376 MHz, D_2O) δ -199.90 – -200.12 (m), -200.24 (dd, *J* = 49.2, 13.7 Hz).

The NMR values matched the reported literature.[3]

HRMS (ESI) m/z calculated for $C_{12}H_{21}FO_{10}$ (M+Na) 367.1, found 367.1.

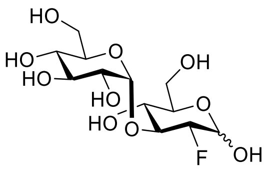

2-deoxy-2-[^{19}F]-fluoro-sakebiose

^{1}H NMR (400 MHz, D_2O) δ 5.37 (d, *J* = 3.9 Hz, 1H), 5.23 (t, *J* = 4.3 Hz, 2H), 4.85 (dd, *J* = 7.9, 2.7 Hz, 1H), 4.44 (ddd, *J* = 48.9, 9.4, 3.9 Hz, 1H), 4.13 (dt, *J* = 53.2, 9.6 Hz, 1H), 4.00 (dt, *J* = 13.1, 10.5 Hz, 1H), 3.87 (m, 1H), 3.83 – 3.57 (m, 15H), 3.46 (m, 3H), 3.35 (t, *J* = 9.4 Hz, 2H).

^{13}C NMR (100 MHz, D_2O) δ 99.15 (C1'α), 99.09 (C1'β), 93.57 (d, *J* = 23.7 Hz, C1β), 91.43 (d, *J* = 185.7 Hz, C2β), 89.64 (d, *J* = 21.7 Hz, C1α), 88.71 (d, *J* = 188.2 Hz, C2α), 79.65 (d, *J* = 17.0 Hz,

C3β), 77.06 (d, J = 17.0 Hz, C3α), 75.75 (C5β), 72.84 (C3'α), 72.83 (C3'β), 71.75 (C5'α), 71.71 (C5'β), 71.51 (C2'α), 71.46 (C2'β), 71.05 (C5α), 69.71 (d, J = 8.0 Hz, C4α), 69.53 (d, J = 7.7 Hz, C4β), 69.31 (C4'α +C4'β), 60.34 - 60.10 (C6α, C6'α, C6β, C6'β).

^{19}F NMR (376 MHz, D_2O) δ -196.49 (dd, J = 50.8, 15.1 Hz), -196.72 (dd, J = 48.9, 13.1 Hz).

HRMS (ESI) m/z calculated for $C_{12}H_{21}FO_{10}$ (M+Na) 367.1, found 367.1.

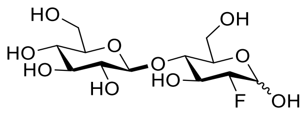

2-deoxy-2[^{19}F]-fluoro-cellobiose

^{1}H NMR (400 MHz, D_2O) δ 5.45 (d, J = 3.9 Hz, 1H), 4.93 (dd, J = 7.8, 2.4 Hz, 1H), 4.54 (d, J = 7.8 Hz, 2H), 4.48 (ddd, J = 49.5, 9.5, 3.9 Hz, 1H), 4.24 – 4.08 (m, 2H), 4.02 – 3.86 (m, 7H), 3.86 – 3.78 (dd, J = 12.4, 5.0 Hz, 1H), 3.78 – 3.69 (m, 4 H), 3.65 (ddd, J = 9.8, 4.9, 2.1 Hz, 1H), 3.58 – 3.38 (m, 6H), 3.33 (m, 2H).

^{13}C NMR (100 MHz, D_2O) δ 102.45 (C1'α+ C1'β), 93.45 (C1β), 93.22 (C2β), 89.90 (C2α), 89.32 (C1α), 77.93 (C4α+C4β), 75.94 (C5'α+C5'β), 75.42 (C3'α+C3'β), 74.86 (C5β), 73.07 (C2'α+C2'β), 72.66 (C3β), 69.92 (C5α), 69.54 (C3α), 69.44 (C4'α+C4'β), 60.56 (C6'α+C6'β), 59.80 (C6β), 59.63 (C6α).

^{19}F NMR (376 MHz, D_2O) δ -199.14 (ddd, J = 51.2, 15.2, 2.3 Hz), -199.33 (dd, J = 49.3, 13.5 Hz). The NMR values matched the reported literature.[4]

HRMS (ESI) m/z calculated for $C_{12}H_{21}FO_{10}$ (M+Na) 367.1, found 367.1.

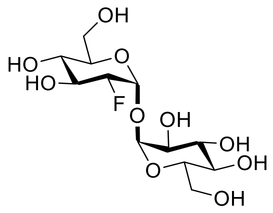

2-deoxy-2[^{19}F]-fluoro-trehalose

^{1}H NMR (400 MHz, D2O) δ 5.44 (d, *J* = 3.9 Hz, 1H), 5.22 (d, *J* = 3.7 Hz, 1H), 4.51 (ddd, *J* = 49.0, 9.6, 3.9 Hz, 1H), 4.13 (dt, *J* = 13.2, 9.4 Hz, 1H), 3.95 – 3.72 (m, 7H), 3.67 (dd, *J* = 9.9, 3.7 Hz, 1H), 3.52 (t, *J* = 9.6 Hz, 1H), 3.46 (t, *J* = 9.4 Hz, 1H).

^{13}C NMR (100 MHz, D_2O) δ 94.00 (C1'), 91.19 (d, *J* = 21.6 Hz, C1), 89.47 (d, *J* = 187.7 Hz, C2), 72.58 (C3'), 72.19 (C5+C5'), 71.08 (d, *J* = 17.4 Hz, C3), 70.93 (C2'), 69.55 (C4'), 69.02 (d, *J* = 8.0 Hz, C4), 60.46 (C6), 60.26 (C6').

^{19}F NMR (376 MHz, D_2O) δ -201.15 (dd, *J* = 49.0, 13.3 Hz).

HRMS (ESI) m/z calculated for $C_{12}H_{21}FO_{10}$ (M+Na) 367.1, found 367.1.

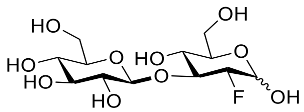

2-deoxy-2[^{19}F]-fluoro-laminaribiose

^{1}H NMR (400 MHz, D_2O) δ 5.46 (d, *J* = 3.8 Hz, 1H), 4.94 (dd, *J* = 7.9, 2.5 Hz, 1H), 4.68 (dd, *J* = 12.1, 8.0 Hz, 2H), 4.61 (ddd, *J* = 49.5, 9.4, 3.9 Hz, 1H), 4.30 (dt, *J* = 53.2, 9.1 Hz, 1H), 4.19 (dt, *J* = 12.6, 9.3 Hz, 1H), 4.09 (dt, *J* = 14.6, 8.7 Hz, 1H), 3.98 – 3.86 (m, 4H), 3.86 – 3.70 (m, 5H), 3.62 – 3.46 (m, 7H), 3.46 – 3.39 (m, 2H), 3.39 – 3.32 (m, 2H).

^{13}C NMR (100 MHz, D_2O) δ 102.45 (C1'α+ C1'β), 93.45 (C1β), 92.87 (C2β), 90.17 (C2α), 89.61 (C1α), 81.65 (C3β), 79.23 (C3α), 75.89 (C5'α+C5'β), 75.70 (C5β), 75.54 (C3'α+C3'β), 73.19 (C2'α+C2'β), 71.08 (C5α), 69.56 (C4'α+C4'β), 67.67 (C4α+C4β), 60.66 (C6'α+C6'β), 60.50 (C6β), 60.28 (C6α).

^{19}F NMR (376 MHz, D_2O) δ -198.87 (dd, *J* = 51.0, 14.6 Hz), -199.20 (dd, *J* = 49.1, 12.7 Hz).

HRMS (ESI) m/z calculated for $C_{12}H_{21}FO_{10}$ (M+Na) 367.1, found 367.1.

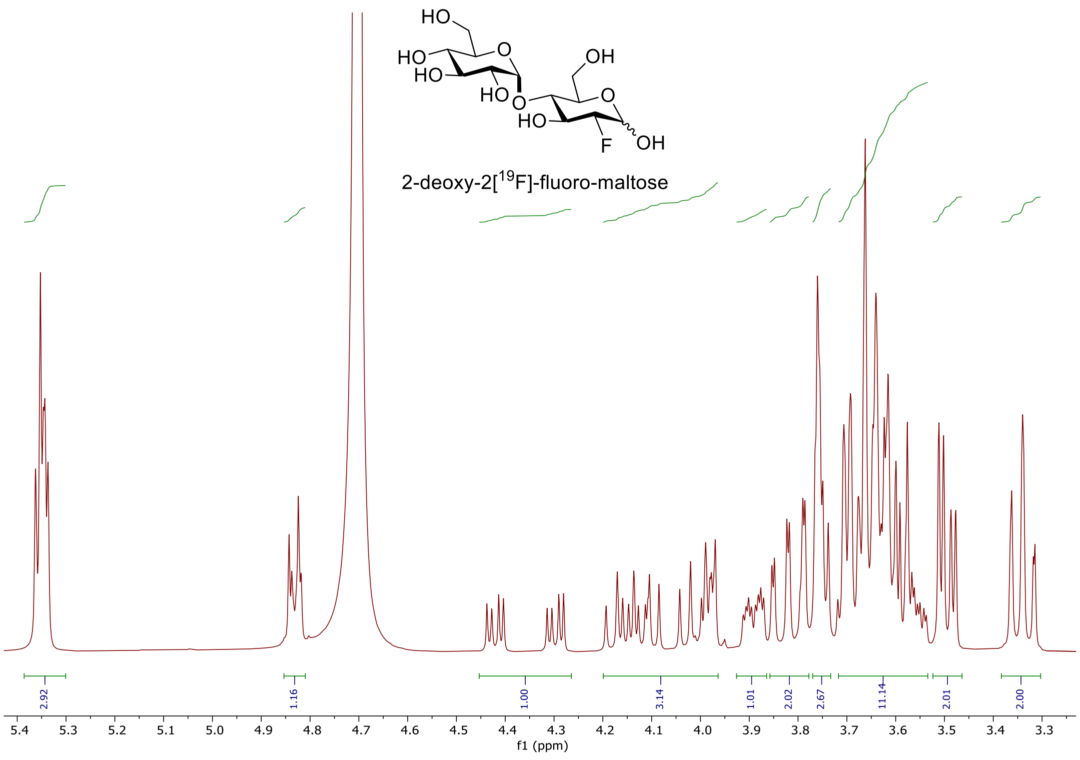

Figure B.3.5. ^{1}H NMR of 2-deoxy-2[^{19}F]-fluoro-maltose in D_2O.

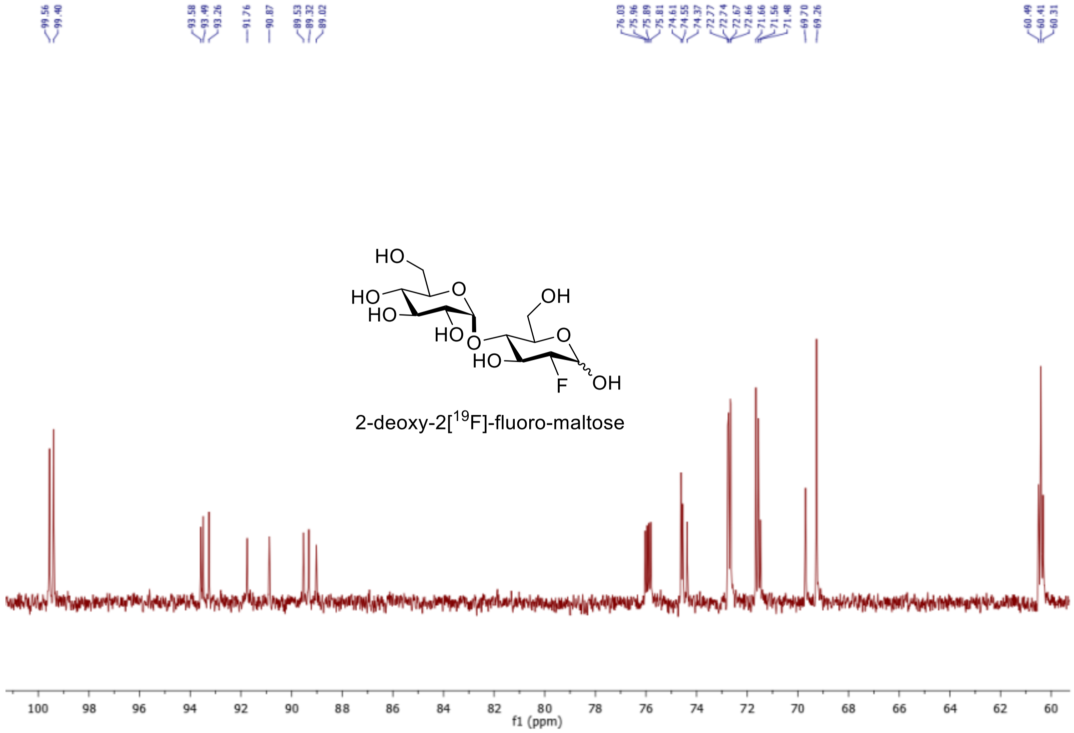

Figure B.3.6. ^{13}C NMR of 2-deoxy-2[^{19}F]-fluoro-maltose in D_2O.

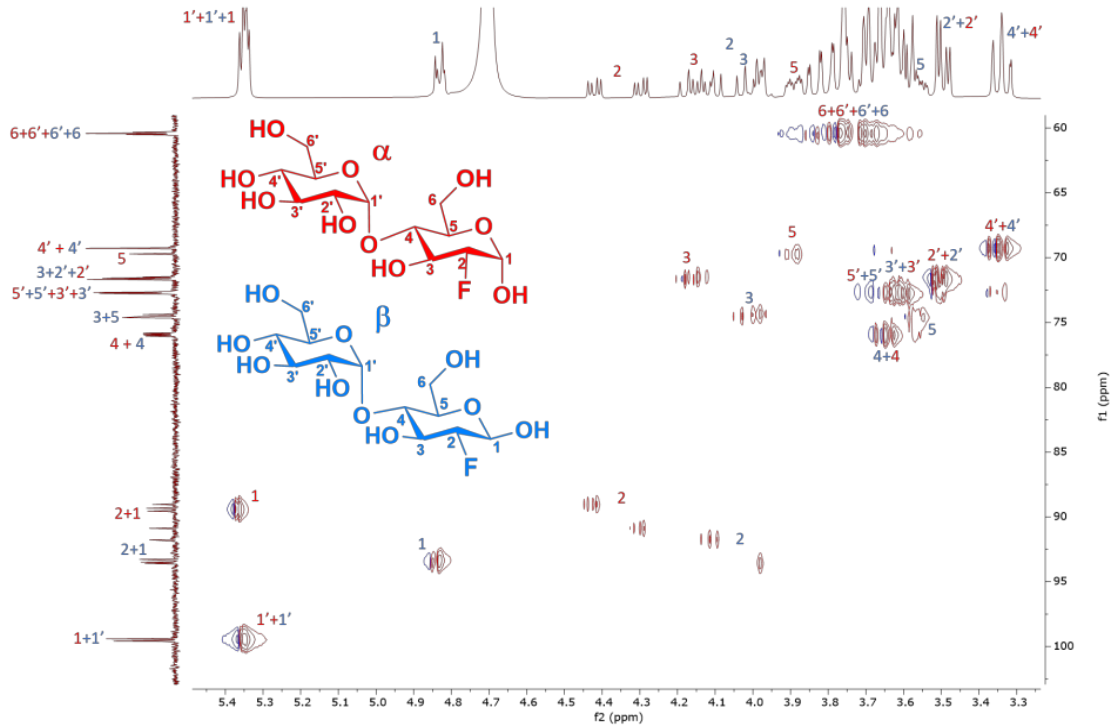

B.3.7. HSQC NMR of 2-deoxy-2[^{19}F]-fluoro-maltose in D_2O.

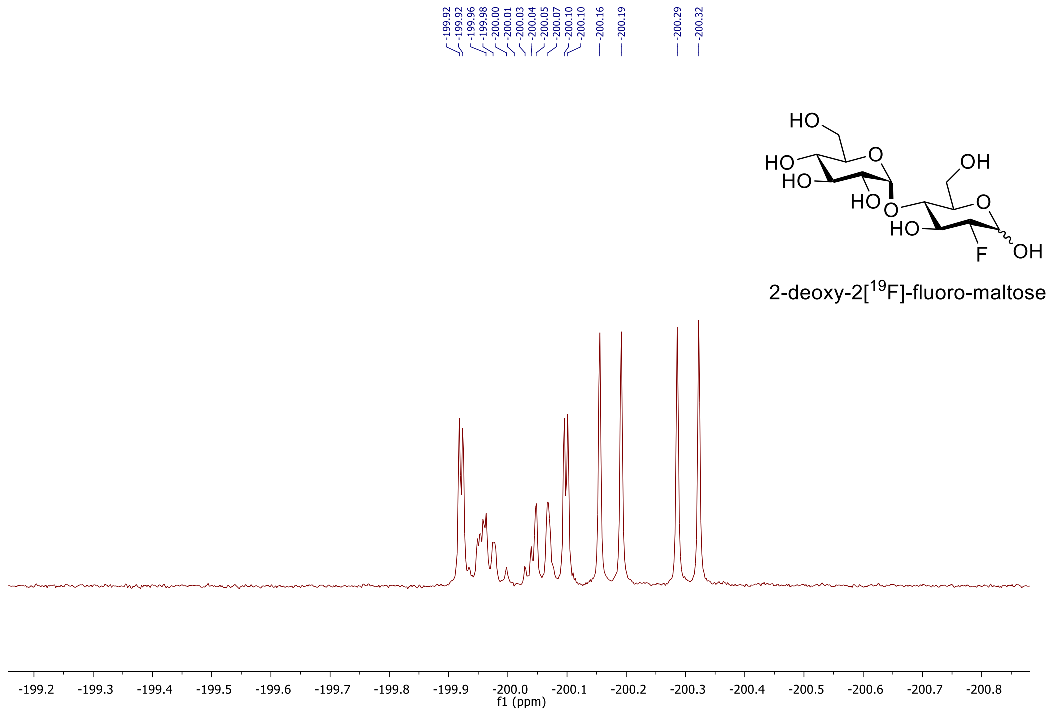

Figure B.3.8. ^{19}F NMR of 2-deoxy-2[^{19}F]-fluoro-maltose in D_2O.

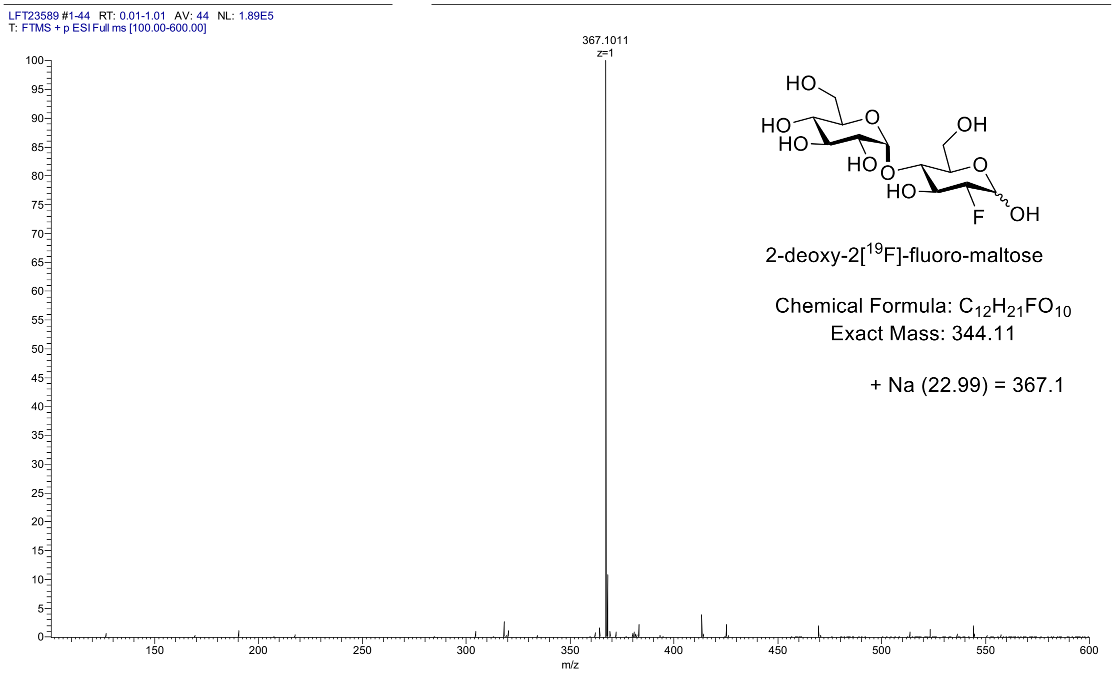

Figure B.3.9. HRMS of 2-deoxy-2[^{19}F]-fluoro-maltose.

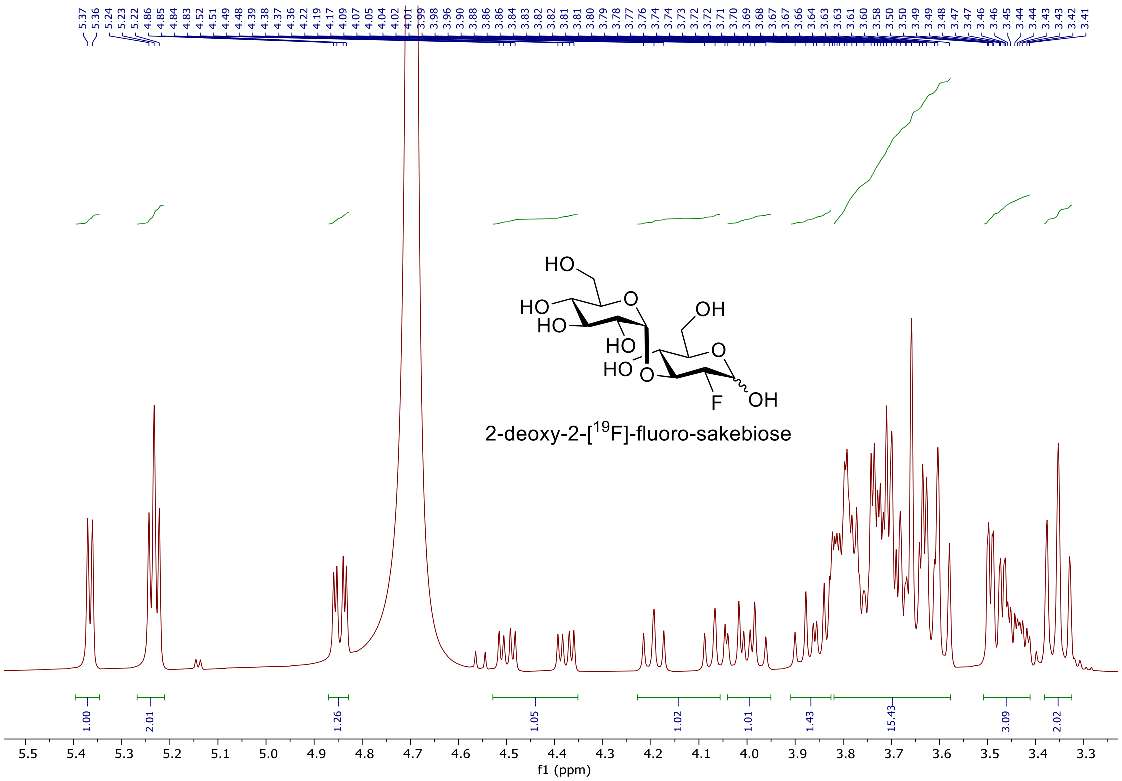

Figure B.3.10. ^{1}H NMR of 2-deoxy-2[^{19}F]-fluoro-sakebiose in D_2O.

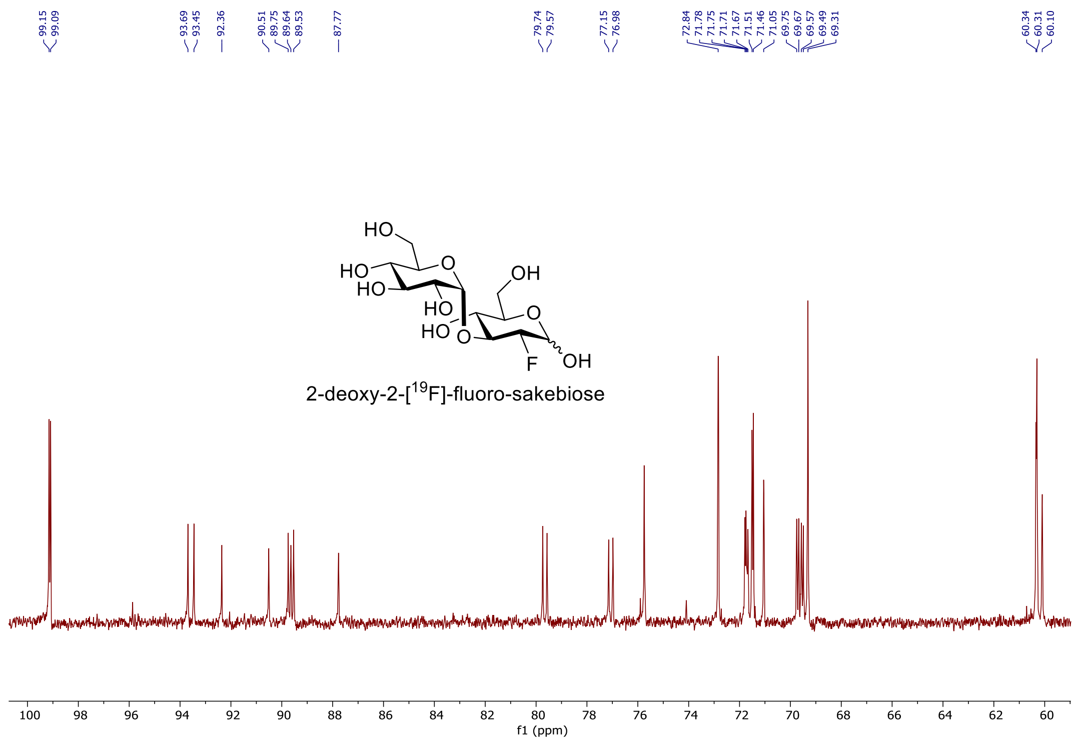

Figure B.3.11. ^{13}C NMR of 2-deoxy-2[^{19}F]-fluoro-sakebiose in D_2O.

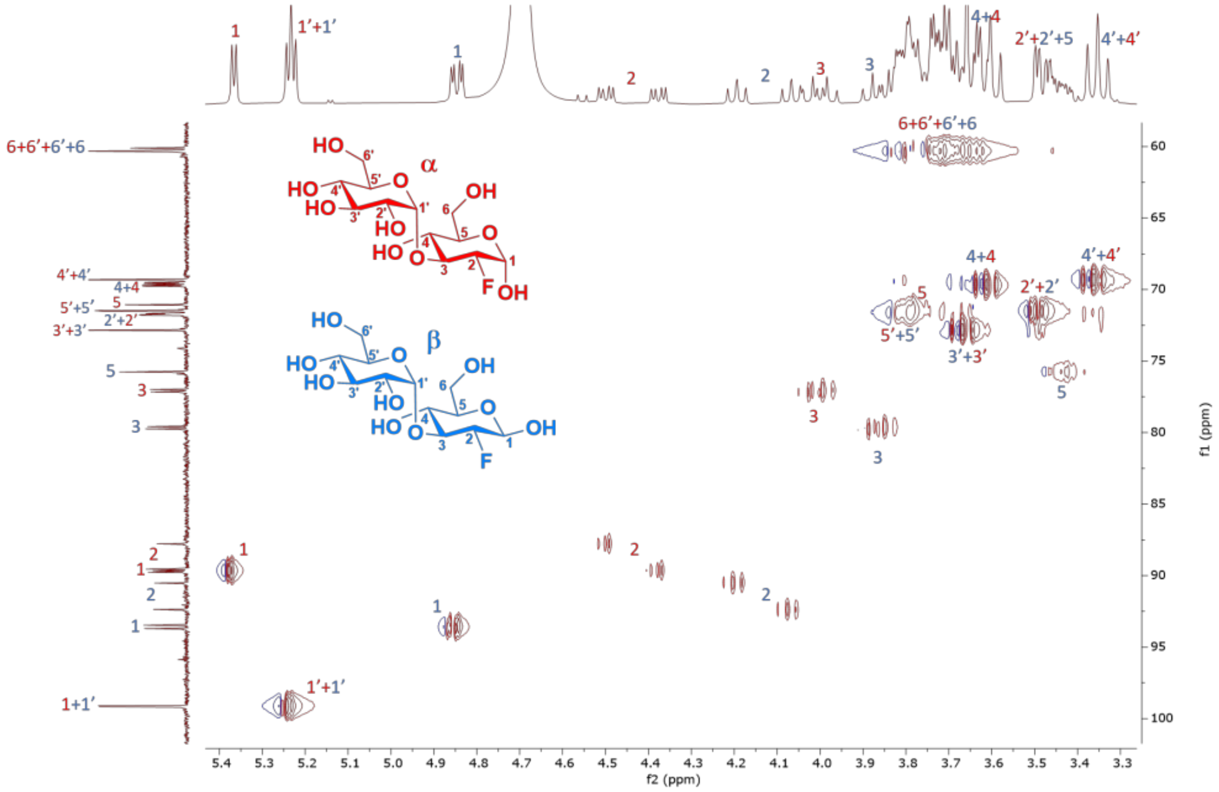

Figure B.3.12. HSQC NMR of 2-deoxy-2[^{19}F]-fluoro-sakebiose in D_2O.

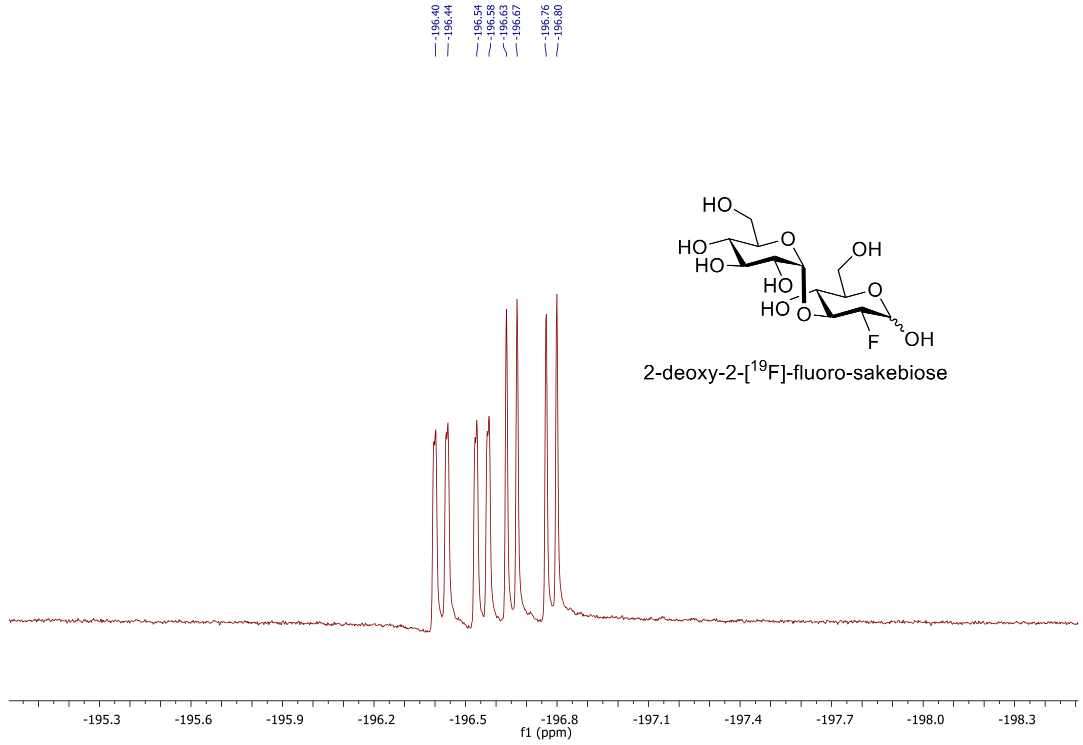

Figure B.3.13. ^{19}F NMR of 2-deoxy-2[^{19}F]-fluoro-sakebiose in D_2O.

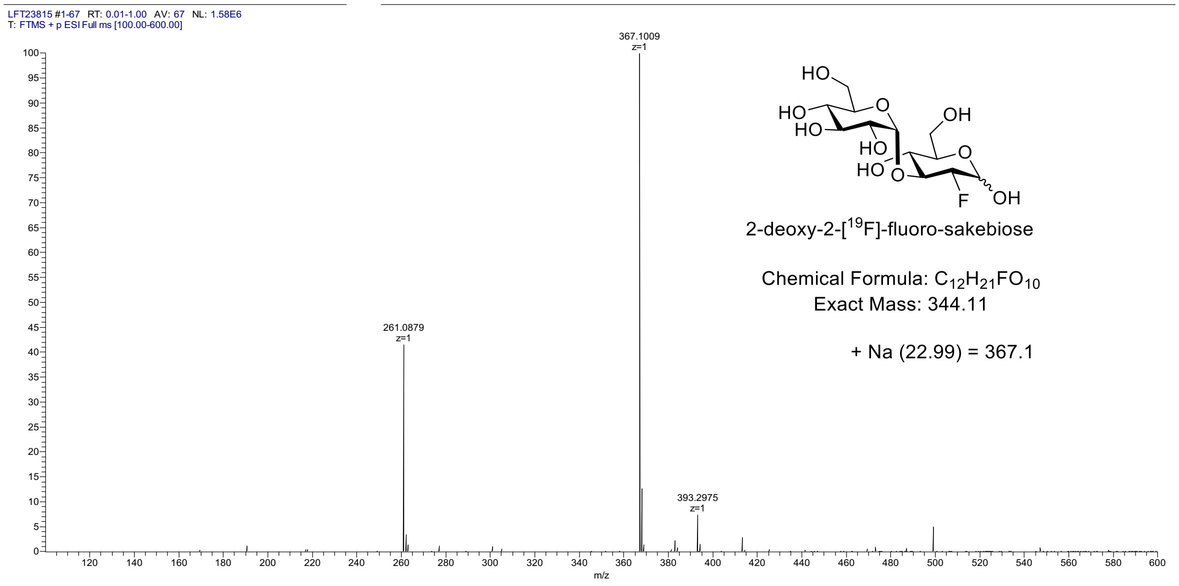

Figure B.3.14. HRMS of 2-deoxy-2[^{19}F]-fluoro-sakebiose.

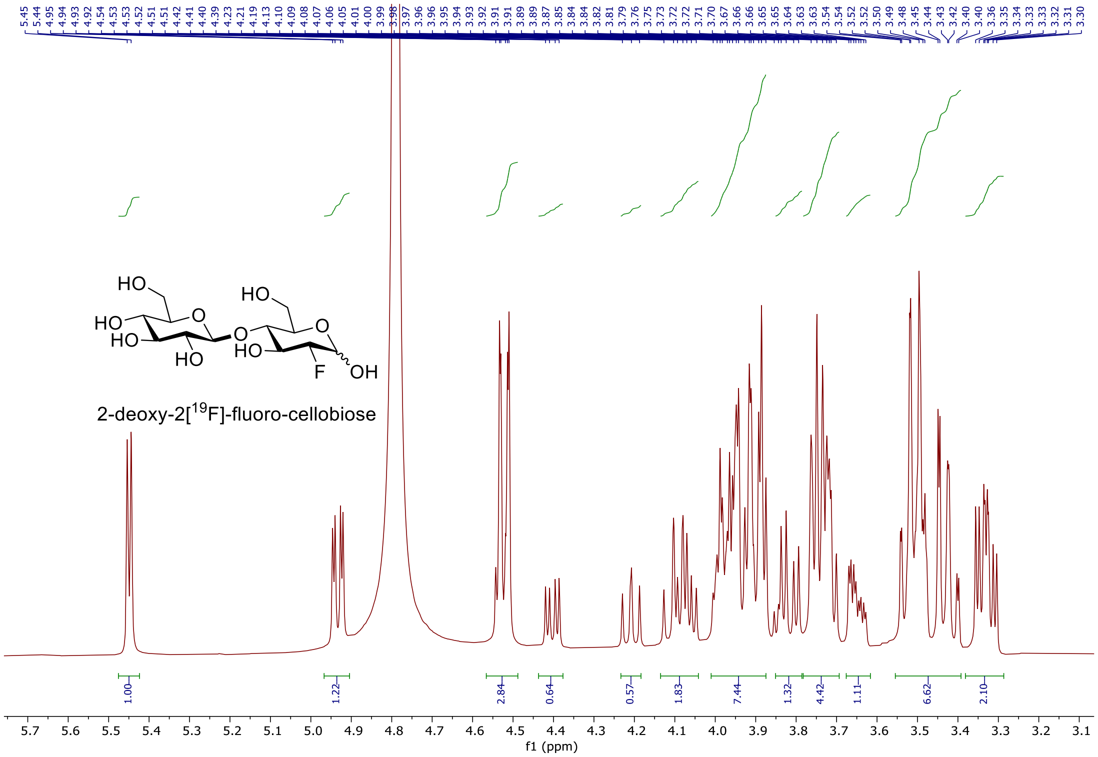

Figure B.3.15. ^{1}H NMR of 2-deoxy-2[^{19}F]-fluoro-cellobiose in D_2O.

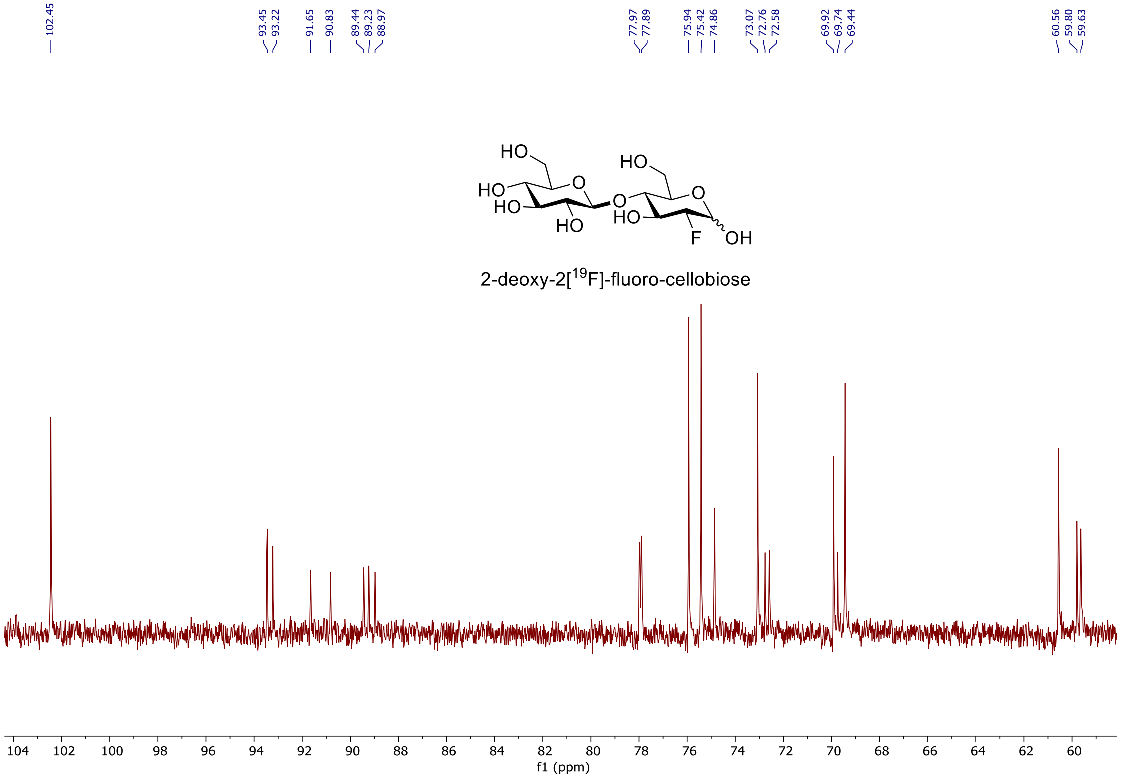

Figure B.3.16. ^{13}C NMR of 2-deoxy-2[^{19}F]-fluoro-cellobiose in D_2O.

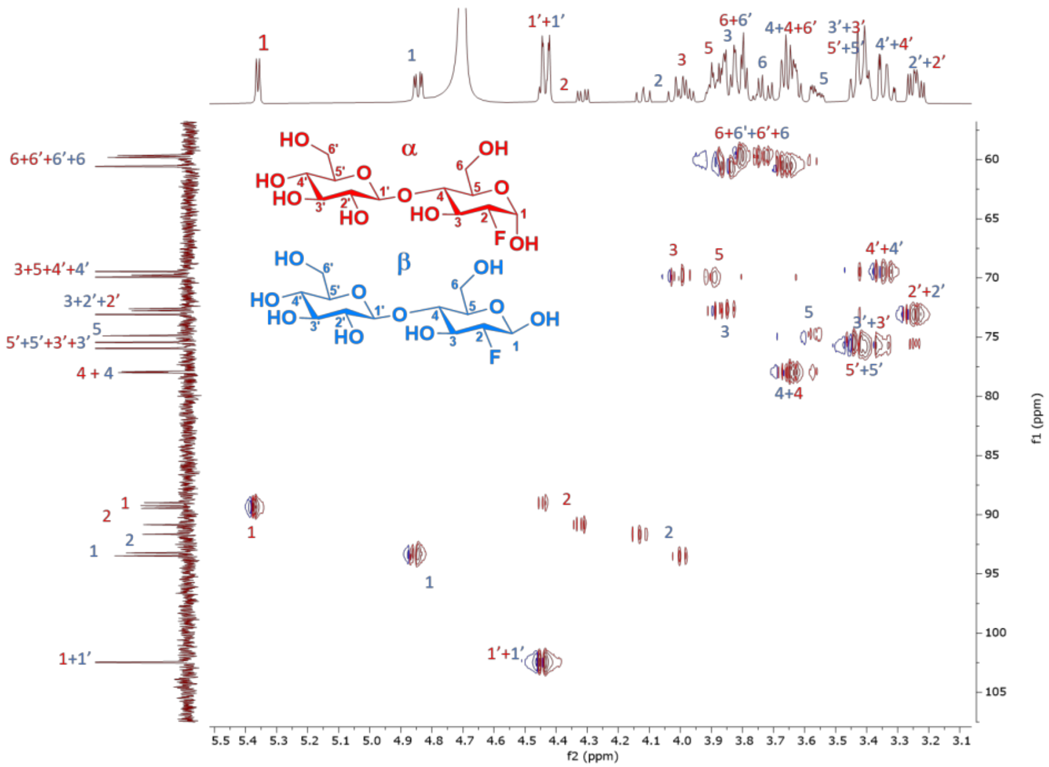

Figure B.3.17. HSQC NMR of 2-deoxy-2[^{19}F]-fluoro-cellobiose in D_2O

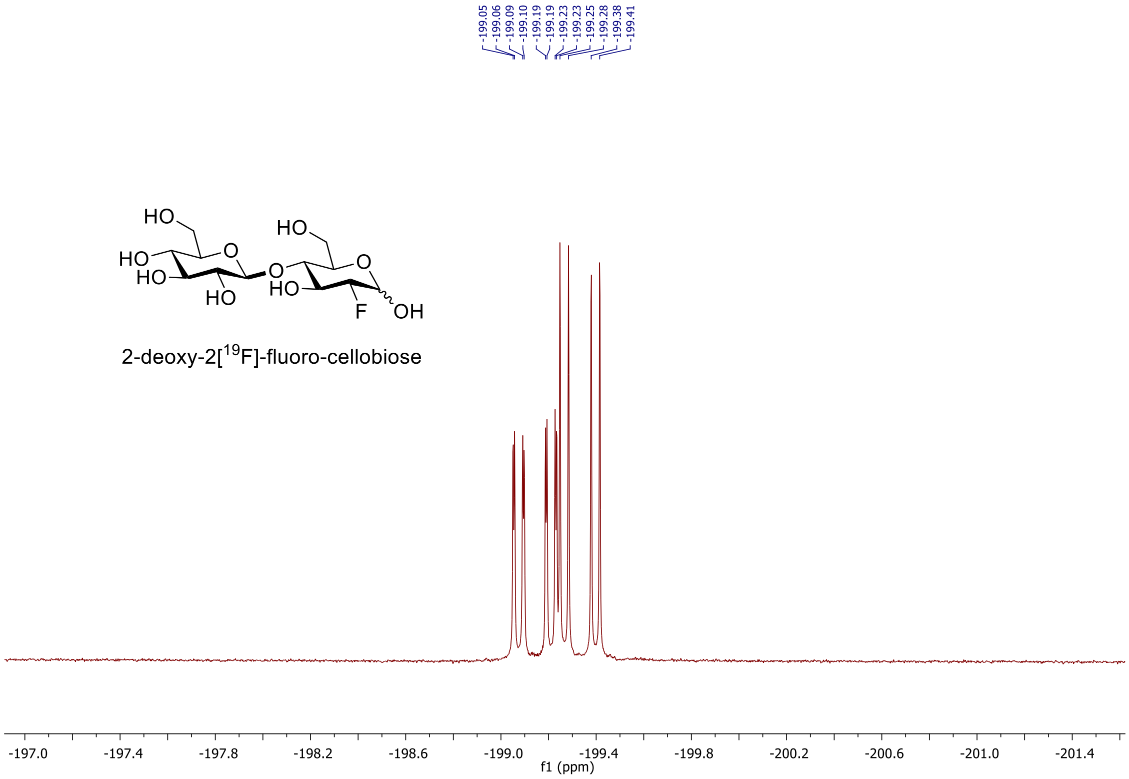

Figure B.3.18. ^{19}F NMR of 2-deoxy-2[^{19}F]-fluoro-cellobiose in D_2O.

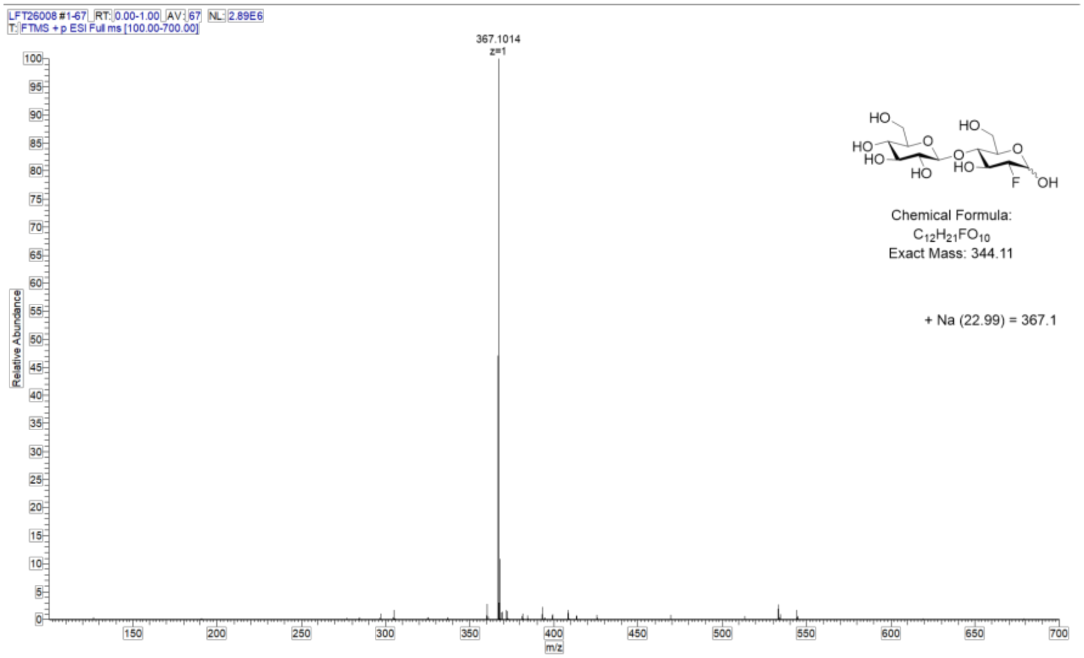

Figure B.3.19. HRMS of 2-deoxy-2[^{19}F]-fluoro-cellobiose.

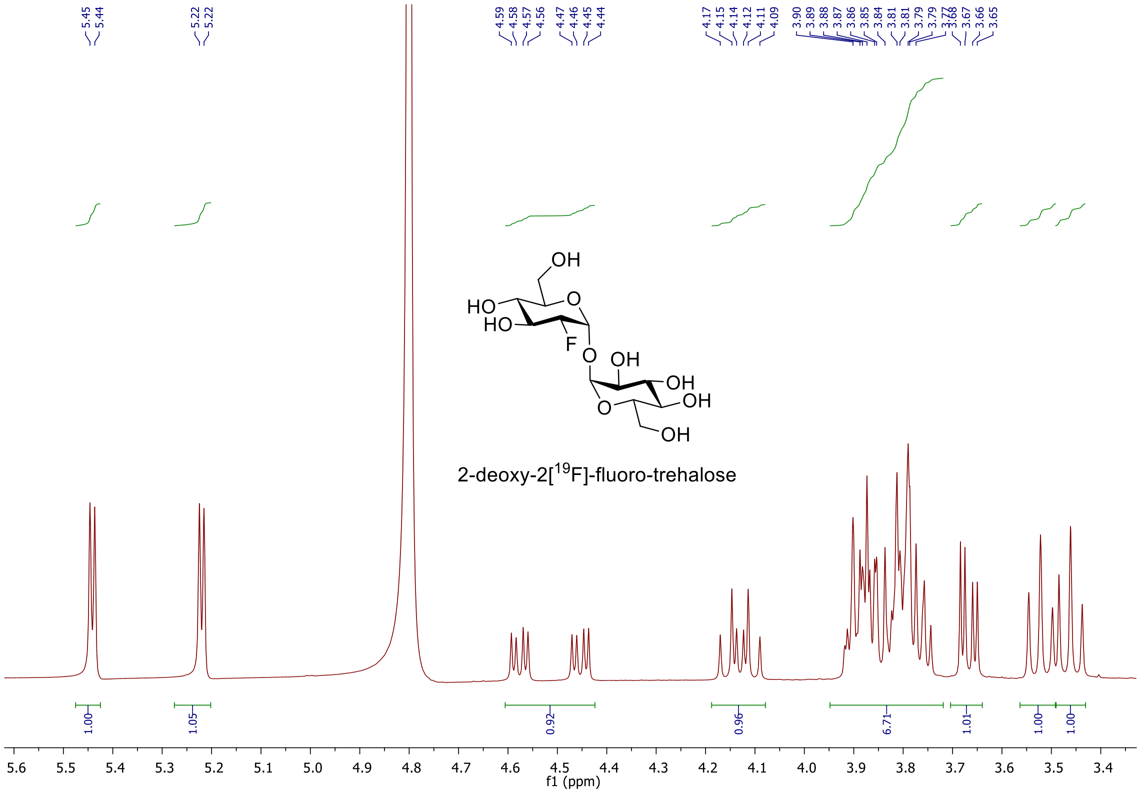

Figure B.3.20. ^{1}H NMR of 2-deoxy-2[^{19}F]-fluoro-trehalose in D_2O.

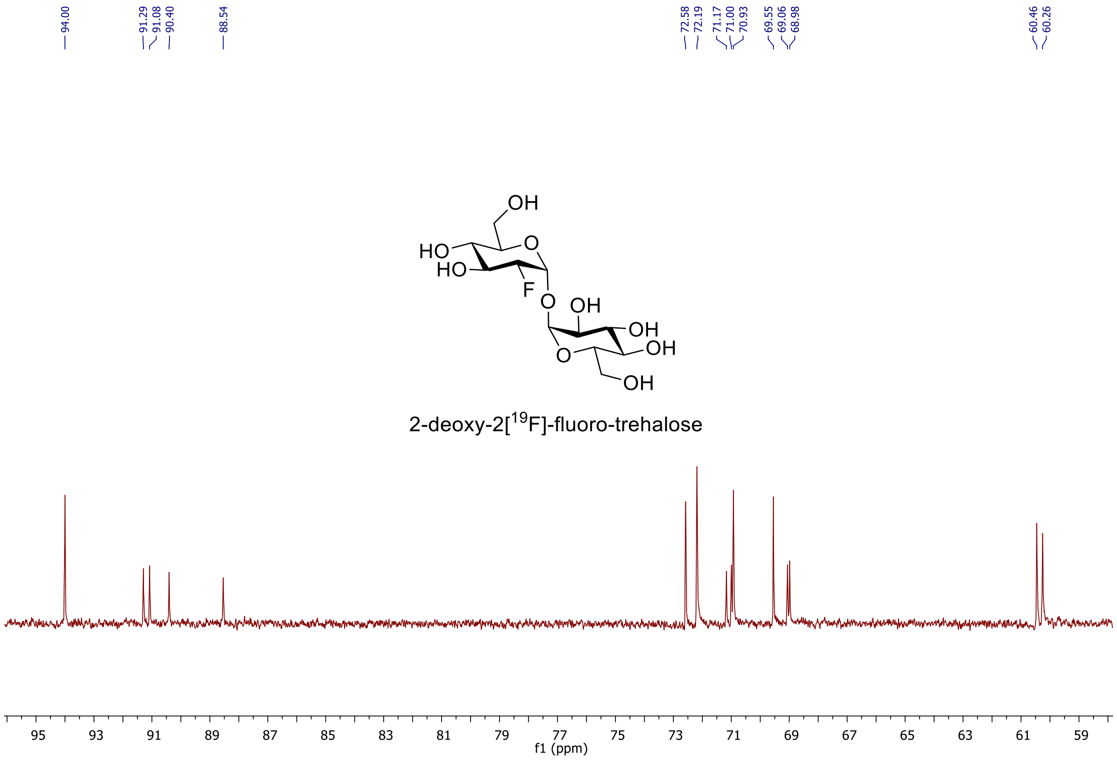

Figure B.3.21. ^{13}C NMR of 2-deoxy-2[^{19}F]-fluoro-trehalose in D_2O.

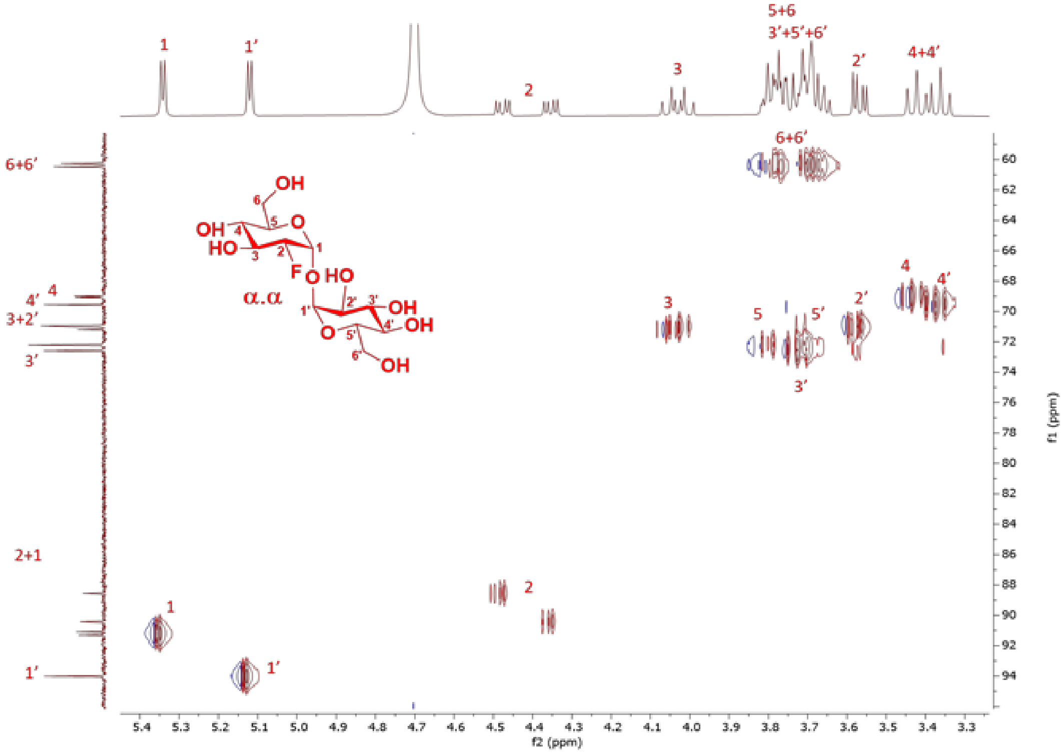

Figure B.3.22. HSQC NMR of 2-deoxy-2[^{19}F]-fluoro-trehalose in D_2O

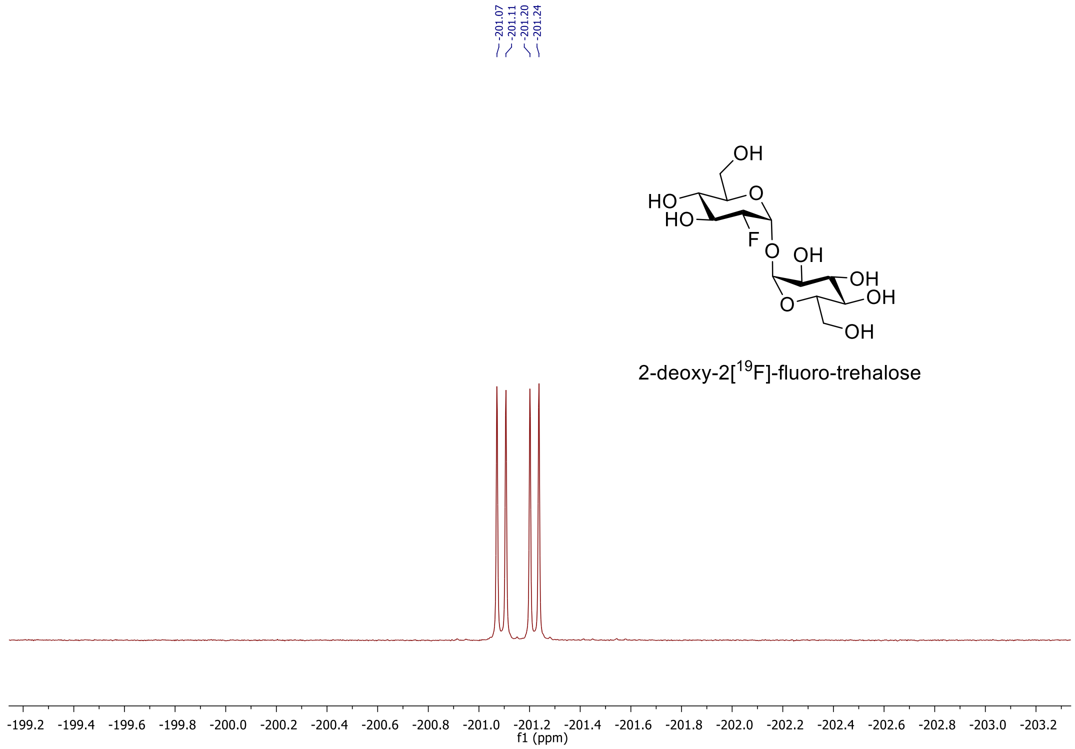

Figure B.3.23. ^{19}F NMR of 2-deoxy-2[^{19}F]-fluoro-trehalose in D_2O.

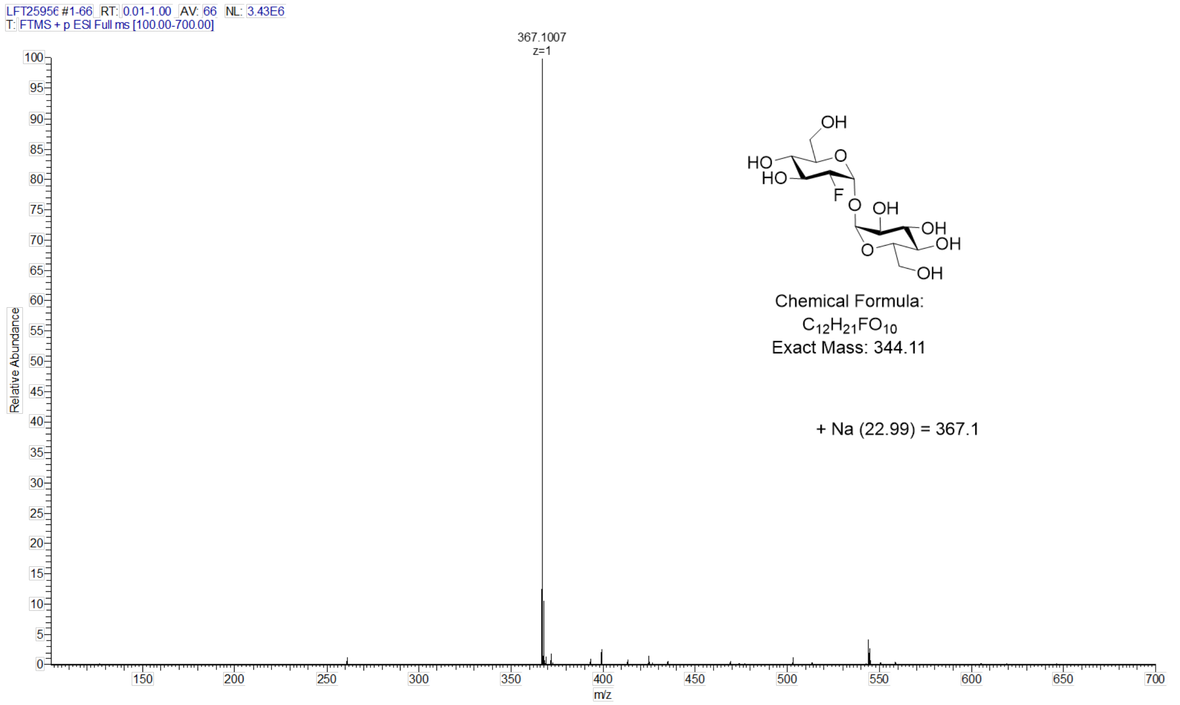

Figure B.3.24. HRMS of 2-deoxy-2[^{19}F]-fluoro-trehalose.

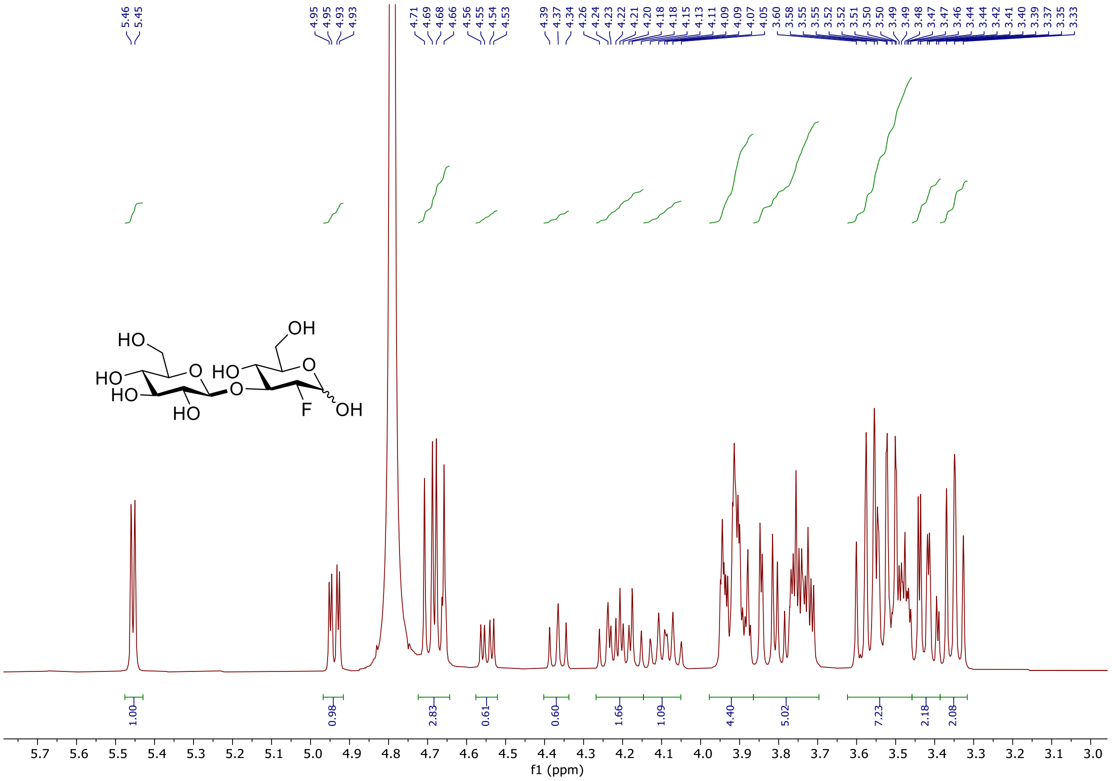

Figure B.3.25. ^{1}H NMR of 2-deoxy-2[^{19}F]-fluoro-laminaribiose in D_2O.

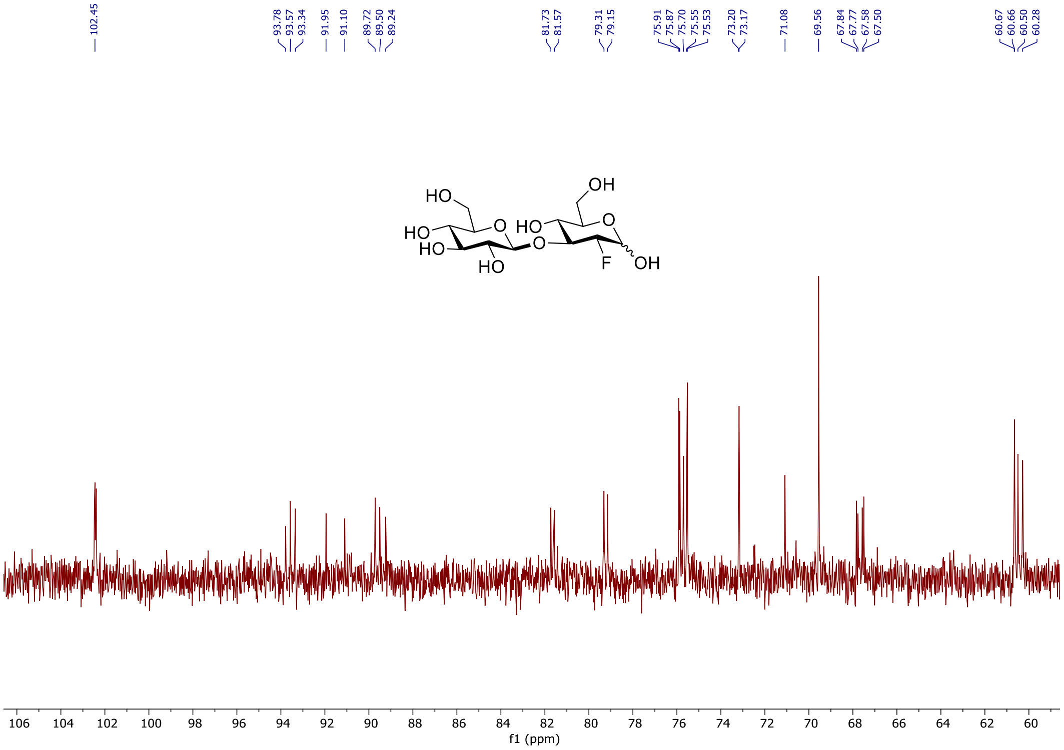

Figure B.3.26. ^{13}C NMR of 2-deoxy-2[^{19}F]-fluoro-laminaribiose in D_2O.

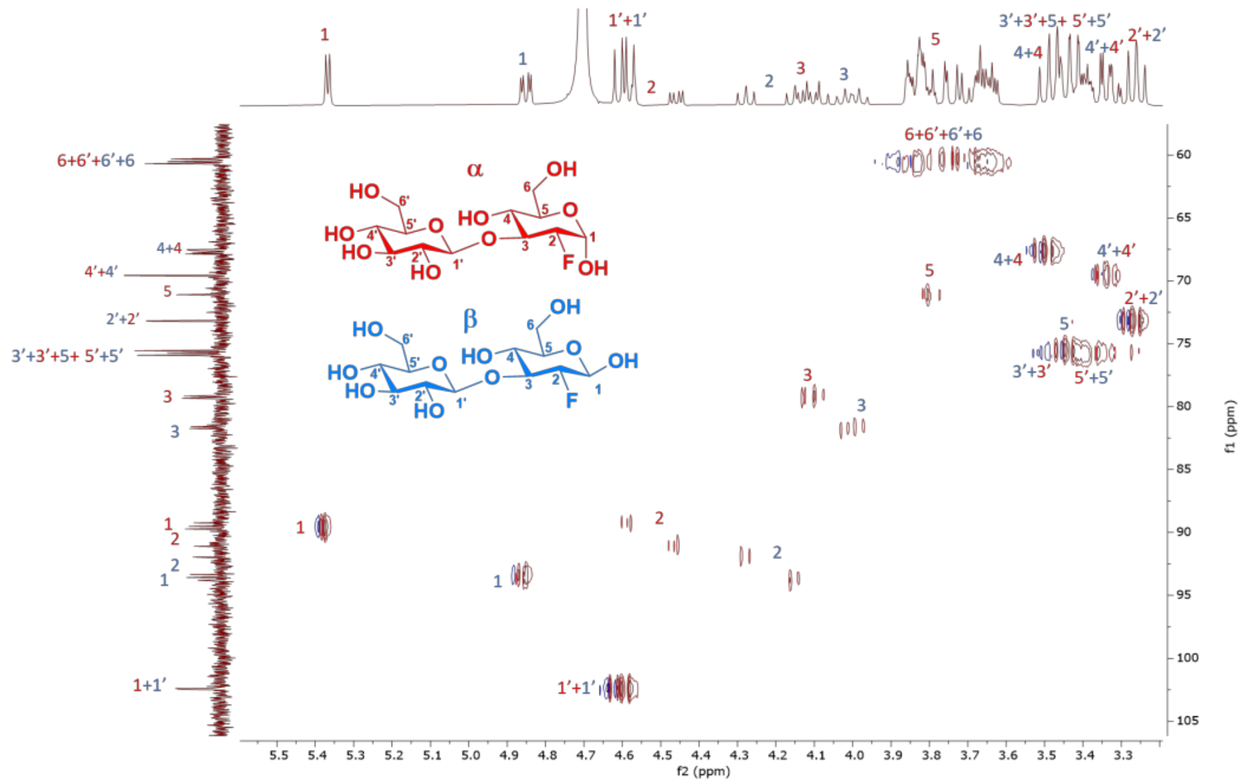

Figure B.3.27. HSQC NMR of 2-deoxy-2[^{19}F]-fluoro-laminaribiose in D_2O.

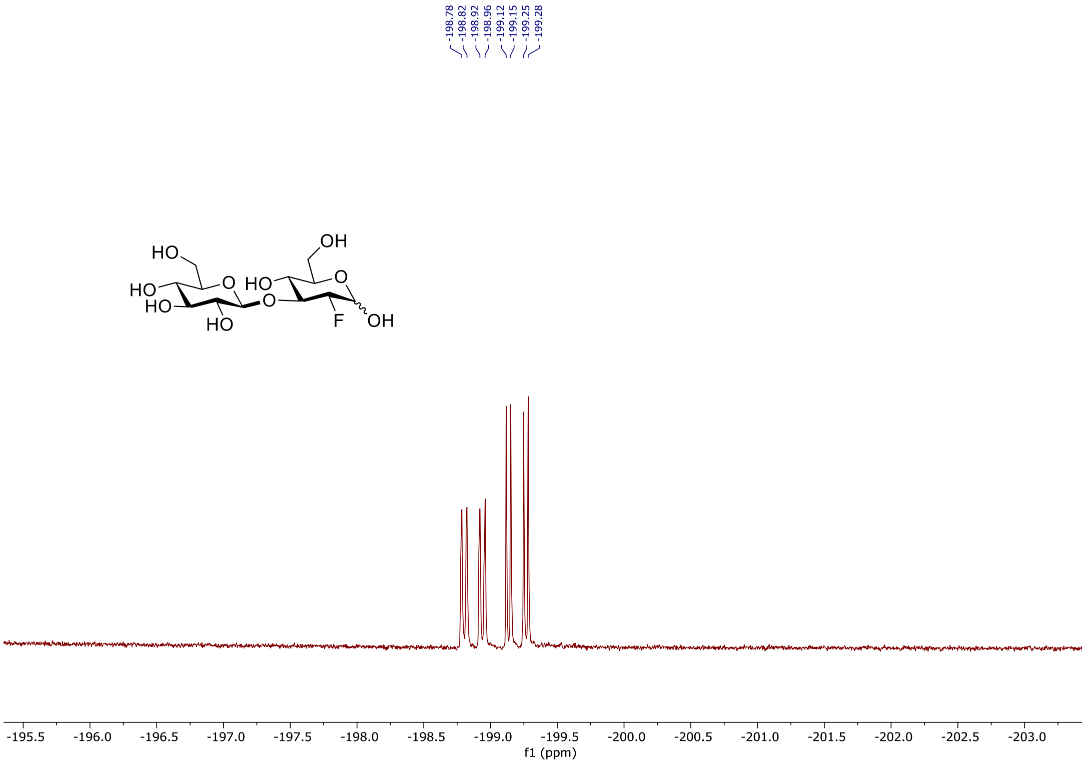

Figure B.3.28. ^{19}F NMR of 2-deoxy-2[^{19}F]-fluoro-laminaribiose in D_2O.

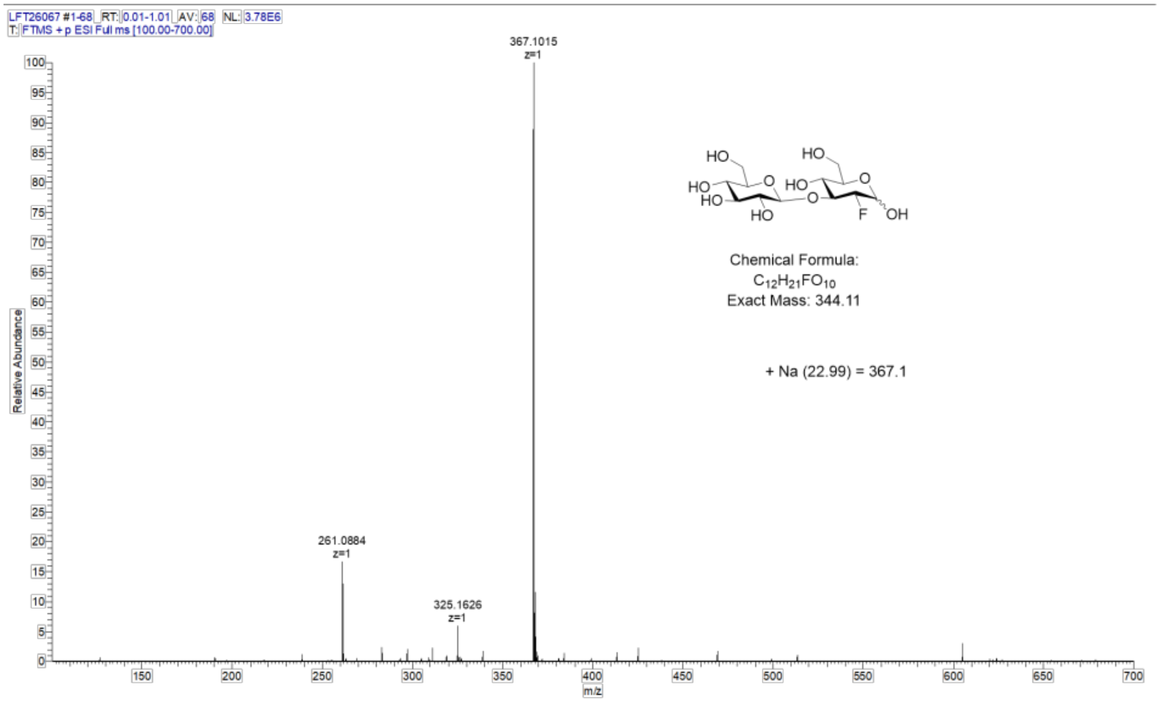

Figure B.3.29. HRMS of 2-deoxy-2[^{19}F]-fluoro-laminaribiose.

B. Radiochemistry

C.1. Radiochemical procedure for 2-deoxy-[^{18}F]-fluoro-D-glucose ([^{18}F]FDG) production:

2-deoxy-[^{18}F]-fluoro-D-glucose ([^{18}F]FDG) was produced in aqueous solution using standard methods.[5]

C.2. Radiochemical procedure for 2-deoxy-[^{18}F]-fluoro-D-sorbitol ([^{18}F]FDS) production:

2-deoxy-[^{18}F]-fluoro-D-sorbitol ([^{18}F]FDS) was synthesized using procedure reported by Weinstein et al.[6]

C.3. Radiosynthesis of 2-deoxy-[^{18}F]-fluoro-maltose ([^{18}F]FDM) and 2-deoxy-2-[^{18}F]-fluoro-sakebiose ([^{18}F]FSK):

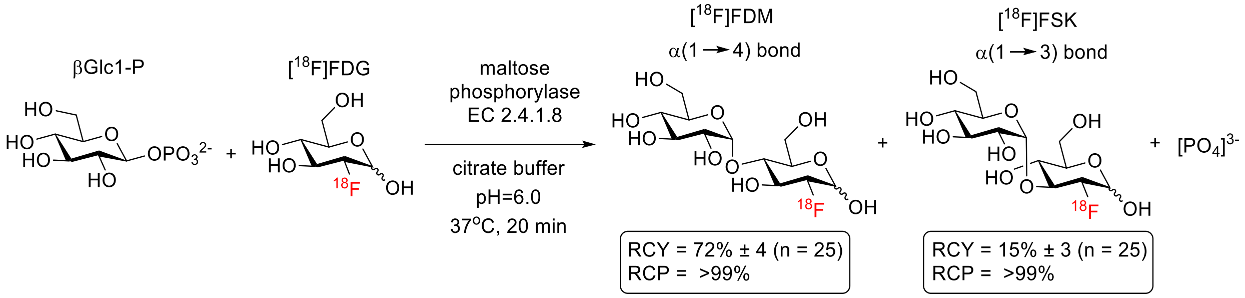

In a 4 mL borosilicate vial containing PTFE stir bar, maltose phosphorylase (EC 2.4.1.8, Sigma Aldrich), (0.3 mg, 3 units) and βGlc1-P (6 mg, 0.020 mmol) were added. A dose of clinical [^{18}F]FDG (10-15 mCi) in citrate buffer (0.1M, pH=6.0, 0.4-0.5 mL) was directly transferred to the vial and the mixture was stirred at 37 °C for 20 min. The mixture was diluted with MeCN then filtered through C18 light cartridge, before being purified via semi prep HPLC (YMC-Pack Polyamine II, 250 X 10 mm) using mobile phase 73% MeCN/27 % H_2O. Both [^{18}F]FDM and [^{18}F]FSK were isolated in 5-7mL fractions. The fractions were then diluted with MeCN (40 mL) before being passed through Sep-pak Plus NH_2 Cartridge at 5 mL/min to trap each dimer product. After flushing the cartridge with air and N_2 gas, they were eluted using saline solution for direct formulation before use in vitro or in vivo. [^{18}F]FDM (RCY= 72% ± 4%, RCP=99%), [^{18}F]FSK (15%± 3%, RCP=99%) (N=25). Chemical purity of both [^{18}F]FDM and [^{18}F]FSK were confirmed by analytical HPLC.

Higher activity reactions were attempted starting with 100-110 mCi of clinical [^{18}F]FDG. Similar yields were obtained for both probes: [^{18}F]FDM (42 ± 3 mCi, RCY= 65% ± 5%, RCP=99%), [^{18}F]FSK (12 ± 1 mCi, 18% ± 2%, RCP=99%) (N=2). The doses obtained for both radiotracers were sufficient for future human studies.

HPLC analysis of [^{18}F]FDM and [^{18}F]FSK

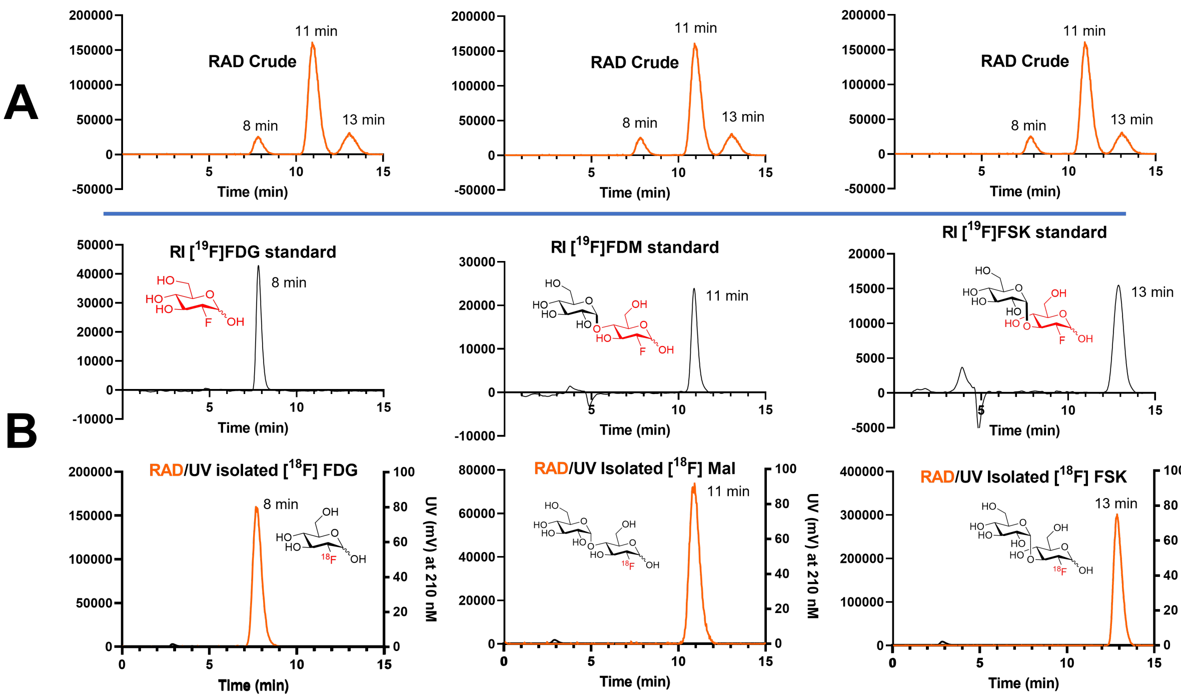

Figure C.3.1. HPLC analysis of 2-deoxy-[^{18}F]-fluoro-maltose ([^{18}F]FDM) and 2-deoxy-2-[^{18}F]-fluoro-sakebiose ([^{18}F]FSK) with YMC-Pack Polyamine II, 250 X 4.6 mm using mobile phase 73% MeCN/27 % H_2O. A) Injection of crude, radioactivity (RAD) detection B) Co-injection of “cold” ^{19}F standard and “hot” ^{18}F isolated tracer, with both refractive index (RI) detection and radioactivity (RAD)/UV detection.

C.4. Radiosynthesis of 2-deoxy-2[^{18}F]-fluoro-cellobiose ([^{18}F]FDC):

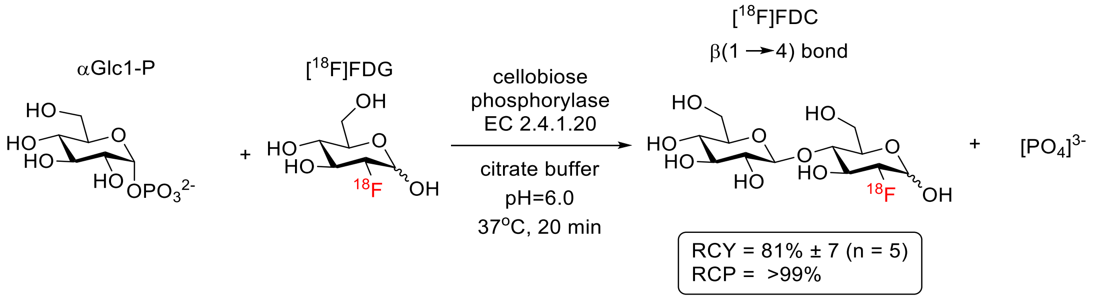

In a 4 mL borosilicate vial containing PTFE stir bar, cellobiose phosphorylase (EC 2.4.1.20, Prof. Bernd Nidetzky lab, Graz University of Technology), (0.3 mg, 3 units) and αGlc1-P (6 mg, 0.020 mmol) were added. A dose of [^{18}F]FDG (10-15 mCi) in citrate buffer (0.1M, pH=6.0, 0.4-0.5 mL) was directly transferred to the vial and the mixture was stirred at 37 °C for 20 min. The mixture was diluted with MeCN then filtered through C18 light cartridge, before being purified via semi prep HPLC (YMC-Pack Polyamine II, 250 X 10 mm) using mobile phase 73% MeCN/27 % H_2O. [^{18}F]FDC was isolated in 5-7mL fractions. The fractions were then diluted with MeCN (40 mL) before being passed through Sep-pak Plus NH_2 Cartridge at 5 mL/min to trap each dimer product. After flushing the cartridge with air and N_2 gas, the tracer was eluted using saline solution for further analysis. [^{18}F]FDC (RCY= 81% ± 7%, RCP=99%), (N=5). Chemical purity of [^{18}F]FDC was confirmed by analytical HPLC.

HPLC analysis of [^{18}F]FDC

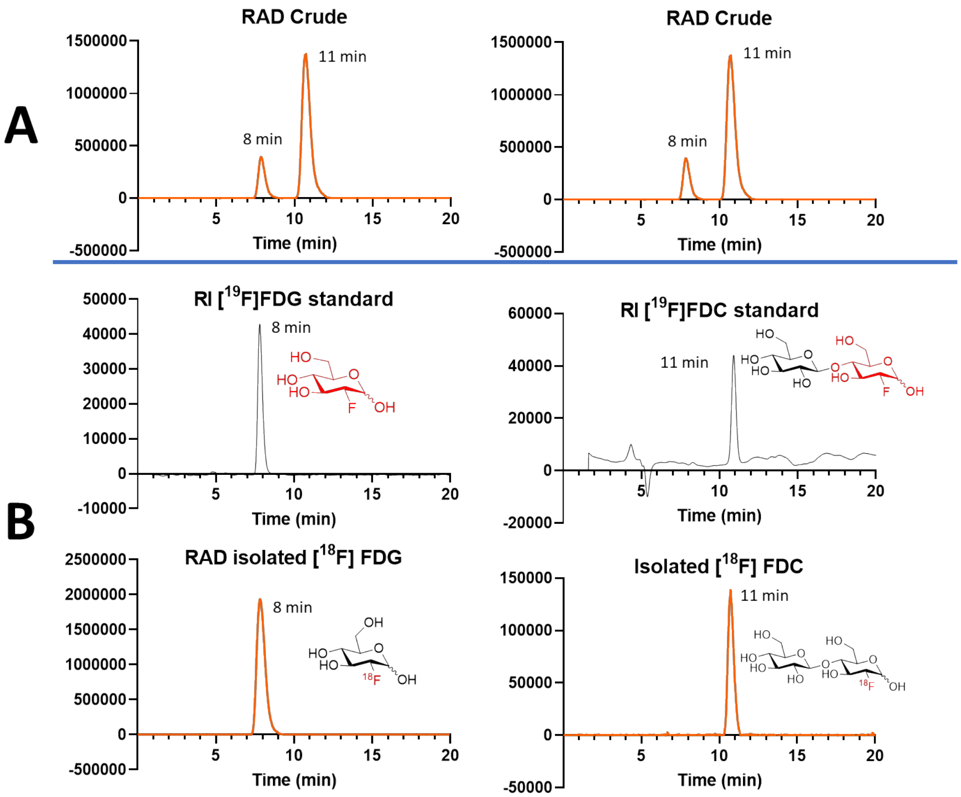

Figure C.4.1. HPLC analysis of 2-deoxy-2[^{18}F]-fluoro-cellobiose ([^{18}F]FDC) with YMC-Pack Polyamine II, 250 X 4.6 mm using mobile phase 73% MeCN/27 % H_2O. A) Injection of crude, radioactivity (RAD) detection B) Co-injection of “cold” ^{19}F standard and “hot” ^{18}F isolated tracer, with both refractive index (RI) detection and radioactivity (RAD) detection.

C.5. Radiosynthesis of 2-deoxy-2[^{18}F]-fluoro-trehalose ([^{18}F]FDT):

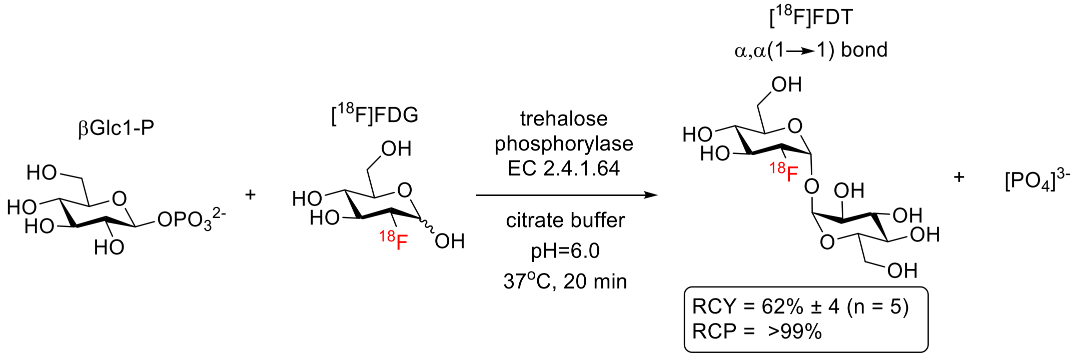

In a 4 mL borosilicate vial containing PTFE stir bar, trehalose phosphorylase (EC 2.4.1.64, Prof. Tom Desmet lab, Ghent University), (0.3 mg, 3 units) and βGlc1-P (6 mg, 0.020 mmol) were added. A dose of [^{18}F]FDG (10-15 mCi) in citrate buffer (0.1M, pH=6.0, 0.4-0.5 mL) was directly transferred to the vial and the mixture was stirred at 37 °C for 20 min. The mixture was diluted with MeCN then filtered through C18 light cartridge, before being purified via semi prep HPLC (YMC-Pack Polyamine II, 250 X 10 mm) using mobile phase 73% MeCN/27 % H_2O. [^{18}F]FDT was isolated in 5-7mL fractions. The fractions were then diluted with MeCN (40 mL) before being passed through Sep-pak Plus NH_2 Cartridge at 5 mL/min to trap each dimer product. After flushing the cartridge with air and N_2 gas, the tracer was eluted using saline solution for further analysis. [^{18}F]FDT (RCY= 62% ± 4%, RCP=99%), (N=5). Chemical purity of [^{18}F]FDT was confirmed by analytical HPLC.

HPLC analysis of [^{18}F]FDT

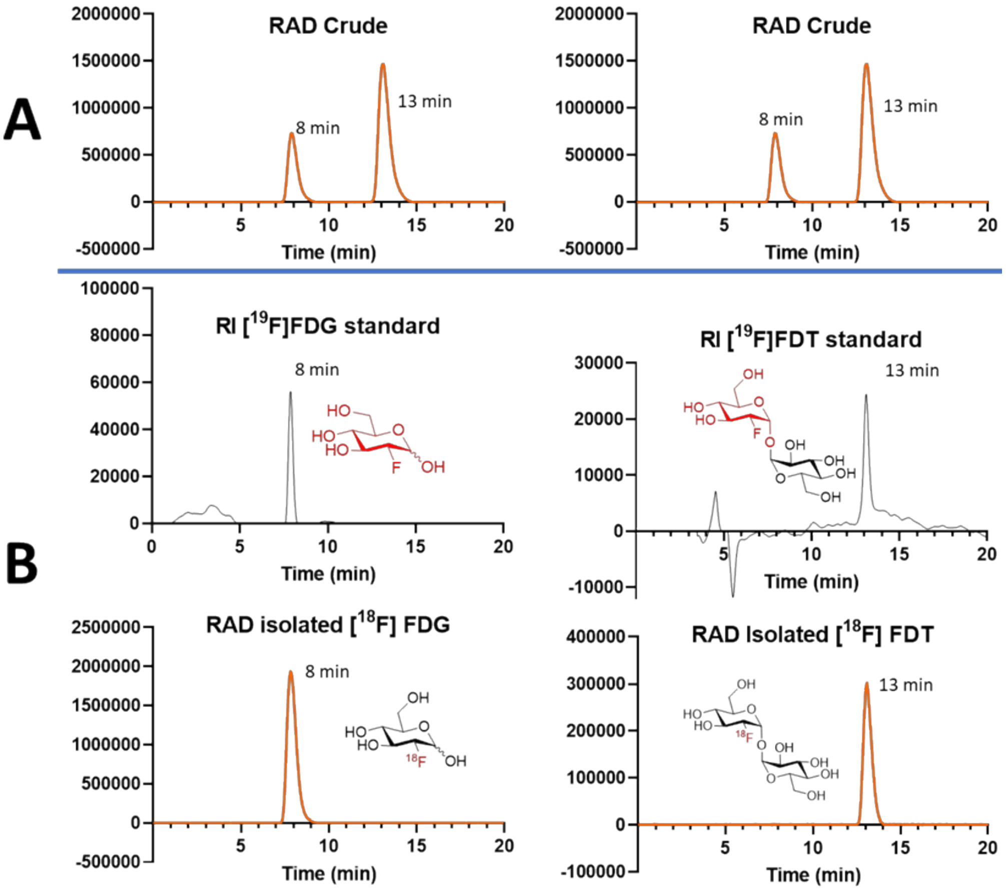

Figure C.5.1. HPLC analysis of 2-deoxy-2[^{18}F]-fluoro-trehalose ([^{18}F]FDT) with YMC-Pack Polyamine II, 250 X 4.6 mm using mobile phase 73% MeCN/27% H_2O. A) Injection of crude, radioactivity (RAD) detection B) Co-injection of "cold" ^{19}F standard and "hot" ^{18}F isolated tracer, with both refractive index (RI) detection and radioactivity (RAD) detection.

C.6. Radiosynthesis of 2-deoxy-2[^{18}F]-fluoro-laminaribiose ([^{18}F]FDL):

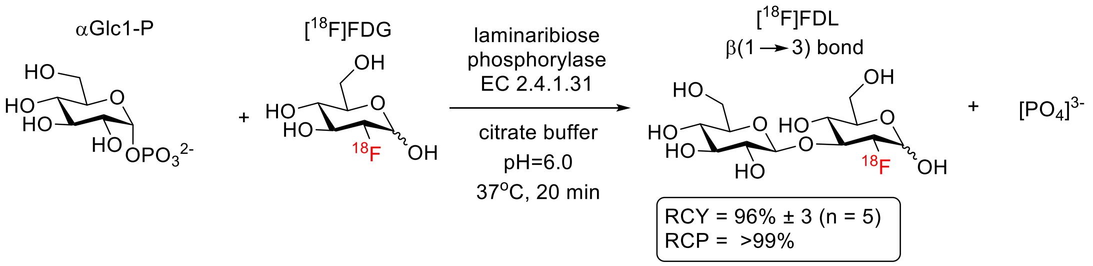

In a 4 mL borosilicate vial containing PTFE stir bar, laminaribiose phosphorylase (EC 2.4.1.31, Prof. Tom Desmet lab, Ghent University), (0.3 mg, 3 units) and αGlc1-P (6 mg, 0.020 mmol) were added. A dose of [^{18}F]FDG (10-15 mCi) in citrate buffer (0.1M, pH=6.0, 0.4-0.5 mL) was directly transferred to the vial and the mixture was stirred at 37 °C for 20 min. The mixture was diluted with MeCN then filtered through C18 light cartridge, before being purified via semi prep HPLC (YMC-Pack Polyamine II, 250 X 10 mm) using mobile phase 73% MeCN/27 % H_2O. [^{18}F]FDL was isolated in 5-7mL fractions. The fractions were then diluted with MeCN (40 mL) before being passed through Sep-pak Plus NH_2 Cartridge at 5 mL/min to trap each dimer product. After flushing the cartridge with air and N_2 gas, the tracer was eluted using saline solution for further analysis. [^{18}F]FDL (RCY= 96% ± 3%, RCP=99%), (N=5). Chemical purity of [^{18}F]FDL was confirmed by analytical HPLC.

HPLC analysis of [^{18}F]FDL

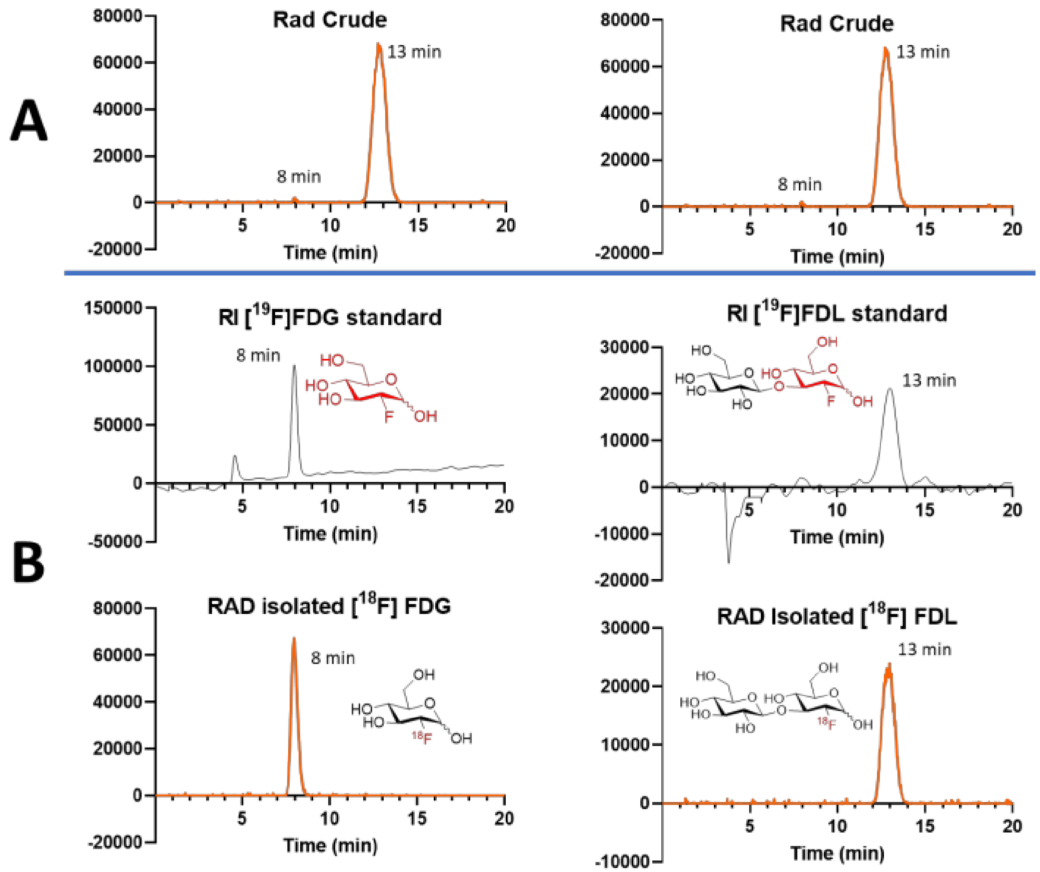

Figure C.6.1. HPLC analysis of 2-deoxy-2[^{18}F]-fluoro-laminaribiose ([^{18}F]FDL) with YMC-Pack Polyamine II, 250 X 4.6 mm using mobile phase 73% MeCN/27 % H_2O. A) Injection of crude, radioactivity (RAD) detection B) Co-injection of “cold” ^{19}F standard and “hot” ^{18}F isolated tracer, with both refractive index (RI) detection and radioactivity (RAD) detection.

C.7. Radiosynthesis of 2-deoxy-2-[^{18}F]-fluoro-sakebiose ([^{18}F]FSK) with sakebiose phosphorylase:

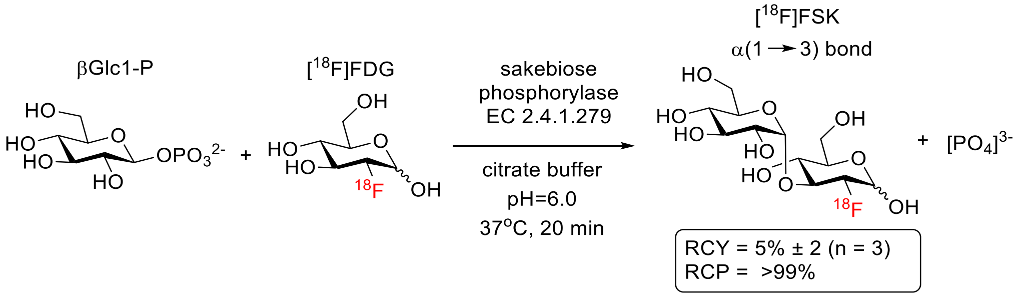

In a 4 mL borosilicate vial containing PTFE stir bar, sakebiose phosphorylase (EC 2.4.1.279, Creative Enzymes), (0.3 mg, 3 units) and βGlc1-P (6 mg, 0.020 mmol) were added. A dose of [^{18}F]FDG (10-15 mCi) in citrate buffer (0.1M, pH=6.0, 0.4-0.5 mL) was directly transferred to the vial and the mixture was stirred at 37 °C for 20 min. The mixture was diluted with MeCN then filtered through C18 light cartridge, before being purified via semi prep HPLC (YMC-Pack Polyamine II, 250 X 10 mm) using mobile phase 73% MeCN/27 % H_2O. [^{18}F]FSK was isolated in 5 mL fraction. The fraction was then diluted with MeCN (40 mL) before being passed through Sep-pak Plus NH_2 Cartridge at 5 mL/min to trap each dimer product. After flushing the cartridge with air and N_2 gas, the tracer was eluted using saline solution for further analysis. [^{18}F]FSK (RCY= 5% ± 2%, RCP=99%), (N=3). Chemical purity of [^{18}F]FSK was confirmed by analytical HPLC.

HPLC analysis of [^{18}F]FSK

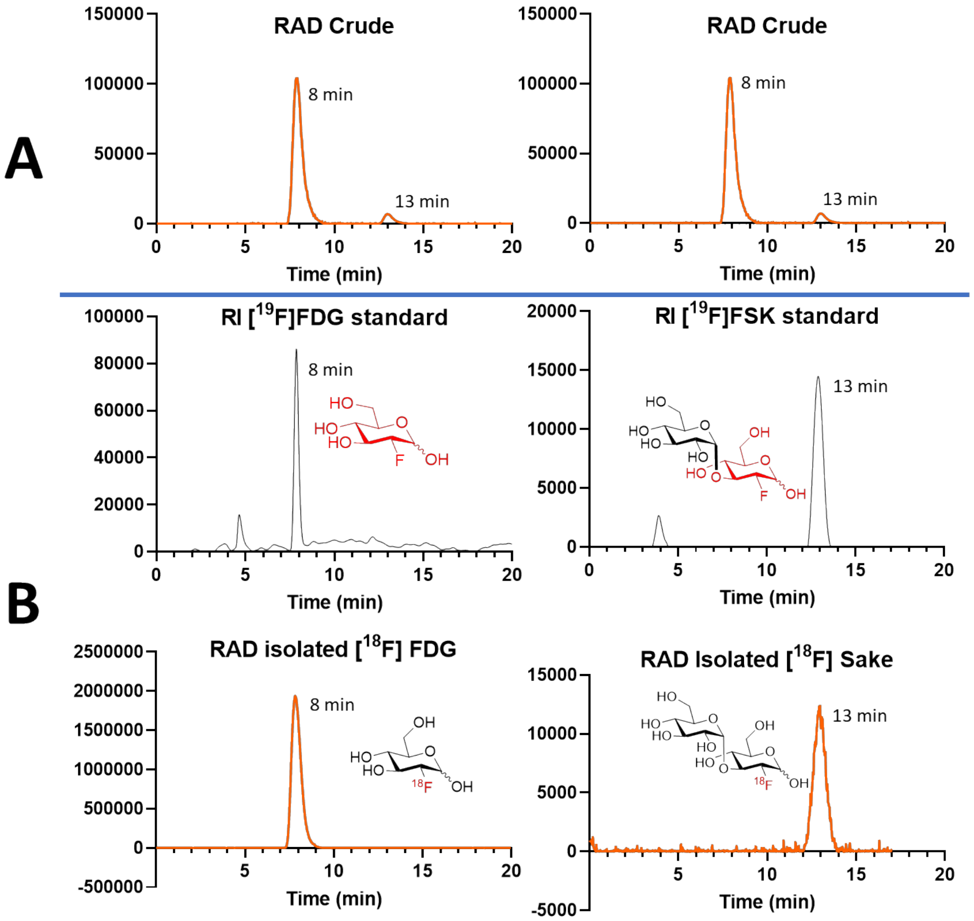

Figure C.7.1. HPLC analysis of 2-deoxy-2-[^{18}F]-fluoro-sakebiose ([^{18}F]FSK) with YMC-Pack Polyamine II, 250 X 4.6 mm using mobile phase 73% MeCN/27 % H_2O. A) Injection of crude, radioactivity (RAD) detection B) Co-injection of "cold" ^{19}F standard and "hot" ^{18}F isolated tracer, with both refractive index (RI) detection and radioactivity (RAD) detection.

C.8. Control Experiment: Reaction without enzyme:

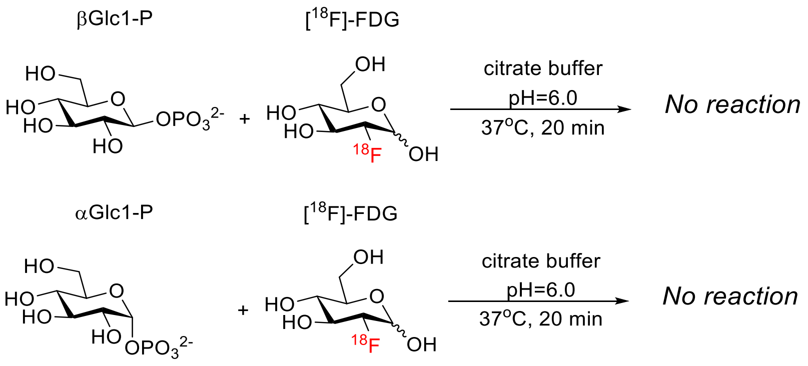

The reaction was conducted with the same conditions except without the presence of phosphorylase.

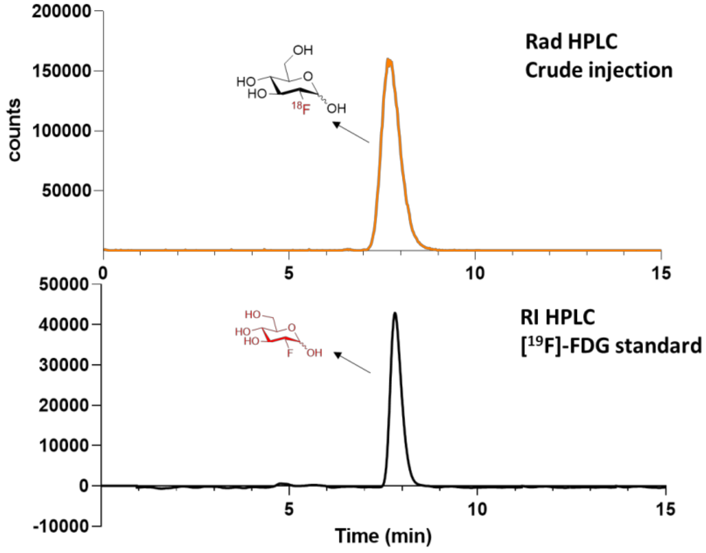

Figure C.8.1. HPLC analysis of control reaction: Rad HPLC only showed presence of unreacted [^{18}F]FDG when phosphorylase was omitted from reaction set up.

C.9. HPLC analysis of precursors, product, and potential by-products for typical phosphorylase catalyzed radiosynthesis

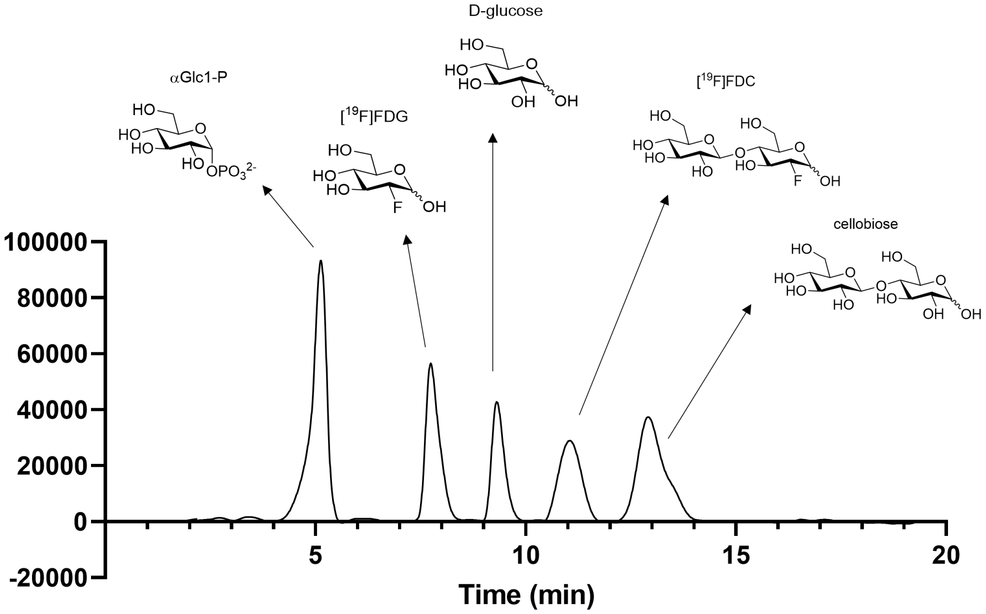

Figure C.9.1. RI HPLC analysis for the Co-injection of α-D-Glc-1-P, "cold" [19F]FDG, D-glucose, "cold" [19F]FDC and cellobiose with YMC-Pack Polyamine II, 250 X 4.6 mm using mobile phase 73% MeCN/27 % H_2O.

This HPLC method shows the separation of all reaction components. The semi prep HPLC that is performed at the end of the phosphorylase catalyzed radiosyntheses using similar method allows isolation of newly formed [^{18}F]-labelled disaccharide product. It helps remove any precursor D-glucose-1-phosphate, [^{18}F]-FDG+glucose and potential "cold" disaccharide formed from final formulation.

C. References

1. Roy, R.; Tropper, F.; Grand-Maître, C. Syntheses of glycosyl phosphates by phase transfer catalysis. *Can. J. Chem.* **1991**, *69*, 1462-1467.

2. Sim, M.; Kondo, H.; Wong, C. Synthesis of dibenzyl glycosyl phosphites using dibenzyl N,N-diethylphosphoramidite as phosphitylating reagent: an effective route to glycosyl phosphates, nucleotides, and glycosides. *J. Am. Chem. Soc.* **1993**, *115*, 2260–2267.

3. Tantanarat, K.; Rejzek, M.; O'Neill, E.; Ruzanski, C.; Hill, L; Fairhurst, S.; Limpaseni, T.; Field, R. An expedient enzymatic route to isomeric 2-, 3- and 6-monodeoxy-monofluoro-maltose derivatives. *Carbohydr. Res.* **2012**, *358*, 12-18.

4. De Andrade P, Muñoz-García JC, Pergolizzi G, Gabrielli V, Nepogodiev SA, Iuga D, Fábián L, Nigmatullin R, Johns MA, Harniman R, Eichhorn SJ, Angulo J, Khimyak YZ, Field RA. Chemoenzymatic Synthesis of Fluorinated Cellodextrins Identifies a New Allomorph for Cellulose-Like Materials *Chem. Eur. J.* **2021**, *27*(4),1374-1382.

5. Hamacher, K., Coenen, H. H., and Stocklin, G. Efficient stereospecific synthesis of no-carrier-added 2-[18F]-fluoro-2-deoxy-D-glucose using aminopolyether supported nucleophilic substitution. *J. Nucl. Med.* **1986**, 27, 235−8.

6. Weinstein, E.A.; Ordonez, A.A.; DeMarco, V.P.; Murawski, A.M.; Pokkali, S.; MacDonald, E.M.; Klunk, M.; Mease, R.C.; Pomper, M.G.; Jain, S.K. Imaging Enterobacteriaceae infection in vivo with 18F-fluorodeoxysorbitol positron emission tomography *Sci Transl Med.* **2014**, *22,* 259.

7. Ludwig F-A, Fischer S, Houska R, Hoepping A, Deuther-Conrad W, Schepmann D, et al. *In vitro* and in vivo Human Metabolism of (S)-[18F]Fluspidine - A Radioligand for Imaging σ1 Receptors With Positron Emission Tomography (PET). Front Pharmacol. **2019** Jun 13;10:534.
